# Emergence of two distinct spatial folds in a pair of plant virus proteins encoded by nested genes

**DOI:** 10.1016/j.jbc.2024.107218

**Published:** 2024-03-24

**Authors:** Esmeralda G. Legarda, Santiago F. Elena, Arcady R. Mushegian

**Affiliations:** 1 Instituto de Biología Integrativa de Sistemas (I2SysBio), CSIC-Universitat de València, Paterna, València, Spain; 2 The Santa Fe Institute, Santa Fe, New Mexico, USA; 3 Division of Molecular and Cellular Biosciences, National Science Foundation, Arlington, Virginia, USA

**Keywords:** genome evolution, movement proteins, overlapping reading frames, virus genomes, convergent evolution of protein structure, tombusvirus

## Abstract

Virus genomes may encode overlapping or nested open reading frames that increase their coding capacity. It is not known whether the constraints on spatial structures of the two encoded proteins limit the evolvability of nested genes. We examine the evolution of a pair of proteins, p22 and p19, encoded by nested genes in plant viruses from the genus *Tombusvirus*. The known structure of p19, a suppressor of RNA silencing, belongs to the RAGNYA fold from the alpha+beta class. The structure of p22, the cell-to-cell movement protein from the 30K family widespread in plant viruses, is predicted with the AlphaFold approach, suggesting a single jelly-roll fold core from the all-beta class, structurally similar to capsid proteins from plant and animal viruses. The nucleotide and codon preferences impose modest constraints on the types of secondary structures encoded in the alternative reading frames, nonetheless allowing for compact, well-ordered folds from different structural classes in two similarly-sized nested proteins. Tombusvirus p22 emerged through radiation of the widespread 30K family, which evolved by duplication of a virus capsid protein early in the evolution of plant viruses, whereas lineage-specific p19 may have emerged by a stepwise increase in the length of the overprinted gene and incremental acquisition of functionally active secondary structure elements by the protein product. This evolution of p19 toward the RAGNYA fold represents one of the first documented examples of protein structure convergence in naturally occurring proteins.

Overlapping, or overprinted, genes are genomic regions that are translated in two alternative reading frames. Overprinted genes are found in all domains of life, but are pervasive in RNA viruses (1, 2, 3, 4, 5, 6, 7). The size and structure of the gene overlaps vary, from the minimal overprinted tetranucleotide AUGA, where the UGA stop codon for the upstream open reading frame (ORF) overlaps with the AUG start codon of the downstream ORF, to the cases when one ORF is entirely embedded in an alternative phase (nested) within another ORF. Gene overprinting is of interest because it offers a distinct mechanism by which new genes may be born, very different from the better-studied processes of intragenomic gene duplication and intergenomic horizontal gene transfer.

In recent years, much work has been done in the bioinformatic characterization of overprinted regions of virus genes (8, 9, 10, 11, 12). The main trends observed on a growing sample of overprinted genes in DNA and RNA viruses are: (*i*) the evolutionarily older, pre-existing ORF (“the parent”) and the younger ORF (“the child”) are usually identifiable within the overprinted pair, primarily on the basis of the broader phylogenetic distribution of the former and more narrow lineage specificity of the latter (13). (*ii*) A pattern of asymmetric evolution is common, in which the nucleotide substitutions in the parent ORF are predominantly synonymous, while the nucleotide substitutions in the child ORF more often cause the amino acid replacements (5). (*iii*) The nucleotide sequences of overprinted genes tend to show the enrichment of cytosines, as well as CC and CG dinucleotides, and depletion of AU, UA and UU dinucleotides (6). Finally, (*iv*) in a large dataset of virus sequences, a mild but statistically significant excess of structurally disordered regions, and of disorder-promoting amino acids R, P and S, is observed in the overlapping compared to the non-overlapping ORFs (10).

Despite these advances in sequence analysis, we still lack understanding of the constraints that may be imposed by gene overprinting at the level of protein three-dimensional structure. Here we report on the sequence and structure evolution of a nested pair of proteins encoded by the members of the *Tombusvirus* genus of plant viruses. Tombusviruses have small icosahedral virions encapsidating the single-strand RNA genome of 4.7 to 4.8 kb. The genome is positive sense and encodes four ORFs: the 5′-proximal ORF encoding RNA replication enzyme (*ORF1/RdRp*) interrupted by a leaky terminator codon and expressed by a readthrough mechanism; the internally located capsid protein ORF expressed from a subgenomic RNA; and two nested genes, *ORF3/p22* and *ORF4/p19*, located in the 3′ region of the genome and both expressed from another subgenomic RNA. See reference (14) and Fig. S1 for the depictions of genome structure and degree of sequence conservation within different ORFs. The *ORF3*/*p22* gene is overprinted in the +2 phase with *ORF4* gene encoding the p19 protein, an RNA-binding protein involved in suppressing the host antiviral RNA silencing response. The structure of p19 in a complex with small RNA, determined using X-ray crystallography (15), belongs to the RAGNYA fold, widely distributed in many RNA-binding and DNA-binding proteins (16). The sequence of p22 belongs to the 30K family of cell-to-cell movement proteins (MPs) found in many plant RNA and DNA viruses. The three-dimensional structure has not been experimentally determined for any member of the 30K family, but recent AlphaFold *in silico* predictions (17) suggest that the 30K family evolved from the virus capsid proteins (CPs) with single jelly-roll fold (18, 19). We studied sequence and protein structure evolution in the p22/p19 overprinted pair to conclude that the same nucleotide sequence may encode two ordered, globular proteins from different structural classes in two alternative reading frames, even as these proteins experience different types of selection pressure.

## Results

### Confirming the predicted single jelly-roll fold in p22 and in other 30K family virus MPs

To infer the spatial fold of the proteins belonging to the 30K family of MPs, we collected a diverse set of 30K homologs from GenBank, including the members of the 30K-related families from the PFAM database (Table S1), as well as the homologs from other virus groups recognized in the literature (19, 20, 21, 22, 23). The PSI-BLAST and HHPred searches allowed us to collect already annotated MP sequences, as well as occasional unannotated homologous domains encoded within longer virus polyproteins. There were very few false positives; one notable exception was a compositionally biased sequence extension in *Emaravirus* MP homologs that led to spurious matches to some bacterial proteins and had to be filtered out (data not shown). The comprehensive sequence set of 30K homologs, used for the structure prediction, included 1730 distinct 30K MP sequences from 728 plant virus species that belong to 37 genera (between 1 and 40 of virus species per genus, supplemented by strains of the same virus species) within 19 families, as well as related MPs from several unclassified viruses.

The MPs from the 30K family that are encoded by different viruses display considerable variation in protein size, from the 21 kDa of *Pelargonium leaf curl virus* (GenPept ID NP_945117) to the 47 kDa in *Suaeda fruticosa betaflexivirus 1* (QQG34646). The alignable region in those diverse proteins is ∼140 amino acids long, constituting about 75% of the length of the shortest proteins. Subsets of the 30K family proteins also include other conserved regions; they may represent structural elaborations of that minimal 140 amino acid core, or sometimes form additional discrete domains, for example, in the case of an aspartic protease domain fused to the 30K MP in genus *Ophiovirus* (24). In this work, we focused on the structural properties shared by all 30K family proteins and did not consider these additional regions.

**Figure 1:**
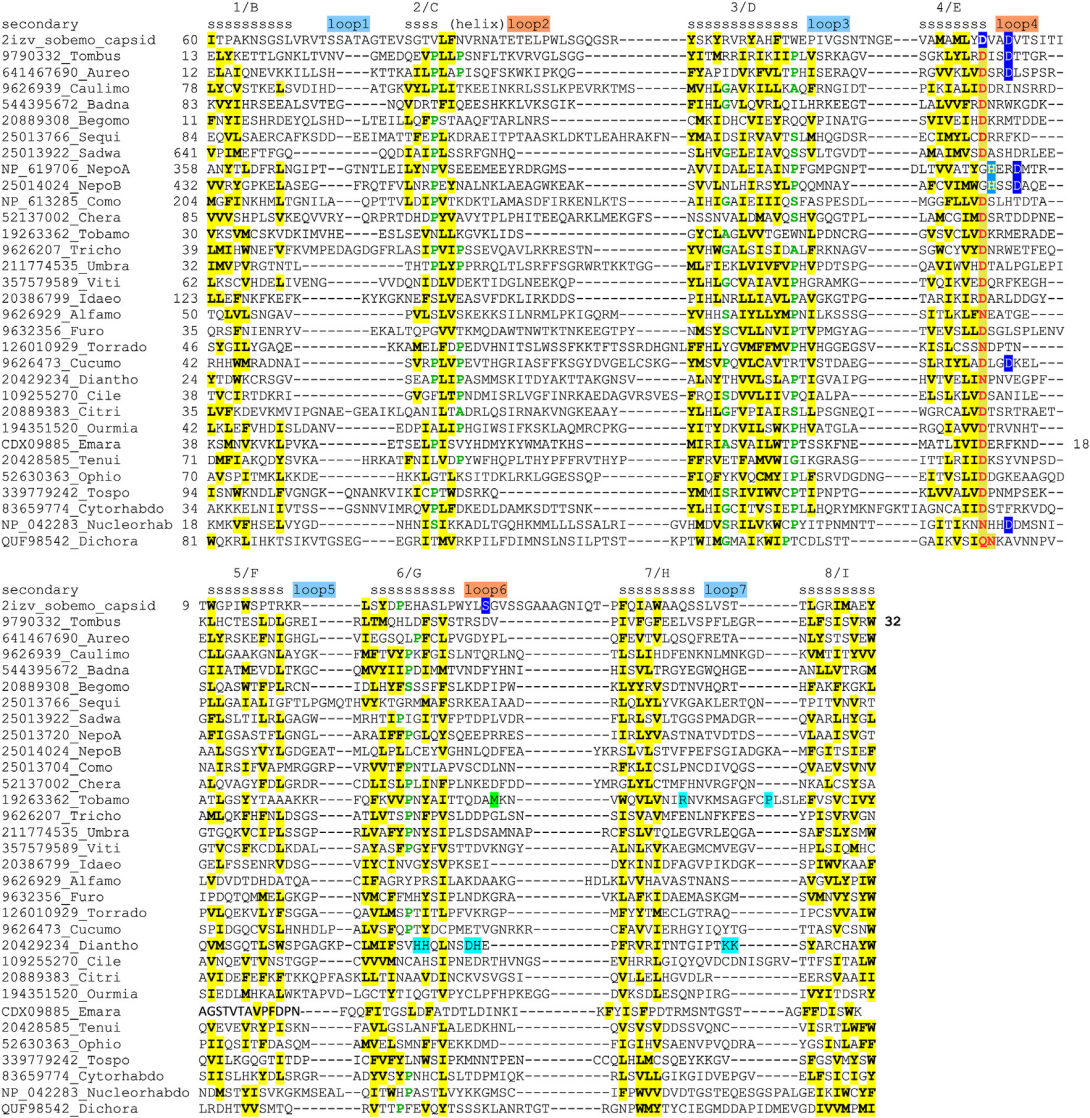
**Multiple sequence alignment of plant virus MPs from the 30K superfamily.** In the *secondarystructure line*, *s* indicates strand, *h* indicates helix; predicted strands and loops are numbered; the strand numbers are followed by the letters commonly used to designate the strands in jelly-roll virus capsid proteins to which 30K family is homologous; the odd-numbered and even-numbered loops are marked by colors to indicate that they are clustered at the opposite ends of the structure. *Yellow shade* indicates bulky hydrophobic amino acid residues (F, I, L, M, V, W, and Y), *red type* indicates acidic residues (D and E), *blue type* indicates basic residues (K and R), and g*reen type* indicates the residues that are likely to induce main chain turns or kinks (A, G, P, and S). Highly conserved D residues in loop 4, potentially involved in cation binding, are shown by the *blue shade*. In *Tobamovirus* and *Dianthovirus* sequences, several residues are highlighted in loops 6 and 7, representing the sites of mutations that impair cell-to-cell movement in the tobacco mosaic virus mutants, in a resistance-breaking strain of tomato mosaic virus, and in the alanine-scanning mutants of red clover necrotic mosaic virus (see text).

Clustering by sequence similarity suggests that the tombusvirus 30K sequences are close to the homologs encoded by viruses in genera *Aureusvirus*, *Dianthovirus*, *Nucleorhabdovirus*, *Ophiovirus*, *Sadwavirus*, and *Vitivirus* (see Fig. S2 for the clustering results and Fig. S3 for an overview of the taxonomic distribution of 30K MP proteins). More detailed phylogenetic analysis of the 30K MP family has been presented in (19). The alignment of representative subset of the 30K-family sequences is shown in Figure 1. As has been noted before (20, 21, 22), the alignments of the 30K family include many conserved hydrophobic residues, in a pattern that suggests seven beta-strands. We now improved the alignment to include eight strands connected with seven loops. There is one highly conserved hydrophilic residue in the entire alignment, the aspartic acid in the previously described “D motif” (20, 21, 22) at the end of strand 4, infrequently replaced by asparagine or, in the case of nepoviruses, by histidine. The D motif has been shown by site-directed mutagenesis experiments to play an essential role in cell-to-cell movement of many groups of viruses (*e.g*., (25, 26, 27)).

**Figure 2:**
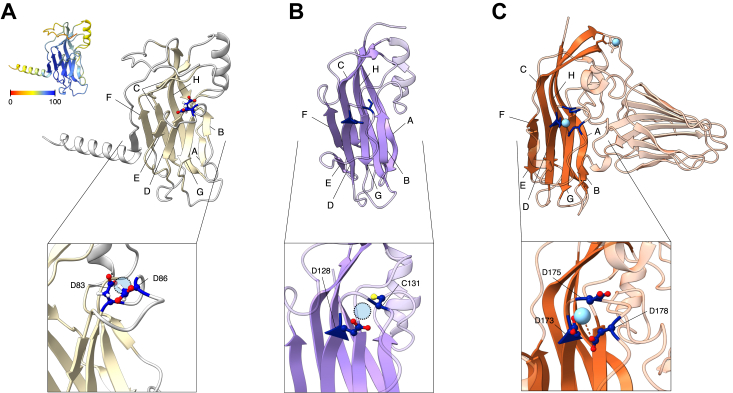
**Known three-dimensional structures of capsid proteins and modeled structure of 30K movement protein.***A*, AlphaFold model of tomato bushy stunt virus p22 protein. *Inset* on the *upper left*, the same molecule is colored according to the pLDDT score of each residue. *B*, monomer subunit of the luteovirus capsid protein (PDB ID 6SCL), the best match in the Dali search of the PDB database with the p22 structure shown in *A*. *C*, monomer of capsid protein of cucumber necrosis virus (PDB ID 4LLF; horizontal/shell domain in a *darker shade*). In the lower row of insets, amino acids that may be involved in coordinating a divalent cation are rendered in *dark blue*, likely positions of a cation are indicated with *light blue circles* (*A* and *B*), and the known position of the Ca^++^ cation is shown by the blue sphere (*C*).

We built the structural model of tombusvirus p22 with the AlphaFold approach, using the multiple alignments of 1730 distinct 30K MP sequences. The models, shown in Figure 2 for tombusviruses and in Fig. S4 for other groups of viruses, have the beta-sandwich supersecondary structure, in which the eight beta-strands are arranged in two four-strand sheets in a mostly antiparallel arrangement. Examination of the quality data for the models of individual p22 proteins from different tombusviruses showed the average confidence level of pLDDT of 83.89 ± 11.80 (±1 SD) for the conserved structural core. The accuracy of the models for the 30K MPs from other plant viruses was similar, with the average pLDDT score 84.22 ± 7.94 (Tables S2 and S3). The pairwise structural similarity between these models occupied a broad range of RMSD values, with median of 7.1 Å (Table S4), but visual inspection confirms that all these models unmistakably belong to the same structure class, with variations in the loop lengths and in occasionally added alpha-helices (Fig. S4). In the course of this work, we noted that structural models have been automatically generated by the RoseTTAFold algorithm (27) for some of the PFAM families comprising the subsets of 30K movement proteins; even more recently, the structures of many of the 30K proteins, predicted by RosettaFold and AlphaFold-family of approaches (27, 28) were discussed in the literature (18, 19). All these predictions and results are fundamentally the same as the results shown here.

Each of the two beta-sheets within the fold consists of antiparallel strands, with the odd-numbered and even-numbered loops located at the opposite ends of the beta-sandwich (Figs. 1 and 2). At the “odd” end, loops 3 and 5 are short and compact, dominated by the residues that are most often associated with kinks and turns in the main chain (A, G, P, and S). Loops 1 and 7 are longer and contain many charged residues. Interestingly, the idea that cell-to-cell spread of infection is a virus-controlled function first came to light after the isolation of two temperature-sensitive mutants of tobacco mosaic virus, Ni2519 and Ls1, which were competent of replication in the primarily infected cells but were unable to spread from them to the neighboring cells at non-permissive temperatures (29, 30). The mutations in both Ni2519 and Ls1 have been mapped to the 30K ORF (31, 32), and, as can be seen in Figure 1, they are located in loop 7. Two of the engineered mutations of *Red clover necrotic mosaic virus* (genus *Dianthovirus*) with impaired cell-to-cell movement (33) also map to this loop. At the “even” end of the sandwich, loop 4 harbors the D motif, and loop 6 is the site of another movement-impairing mutation in dianthovirus MPs, as well as the resistance-breaking mutations in the tomato strains of *Tobacco mosaic virus* (33, 34).

We compared the models of MPs from the 30K family to the database of known 3D structures of proteins using the DALI approach (35). All models were most similar to proteins with the jelly-roll beta-sandwich topology, with the highest similarity to the single jelly-roll domains of the CPs of various small icosahedral DNA and RNA viruses, in agreement with the recent reports by Hak *et al.* (17) and Butković *et al.* (19) (Figs. 2 and S4 and Table S2). When the matching virus capsid structure contained two jelly-roll units, the match almost always was to the horizontal/shell domain (Fig. 2). In several CPs, three D residues (D173, D175, and D178 in Fig. 2*C*) are thought to be involved in the coordination of divalent cations, which may play a role in stabilizing the trimers within a capsomer (36, 37, 38). The first two of those D residues superimpose on the D83 and D86 of p22, where D83 corresponds to the highly conserved D motif in the 30K movement proteins.

In the course of this analysis, we also revisited the sequence-structure relationships of putative MP of nucleorhabdoviruses and dichoraviruses (gene 3 products, or Sc4 protein in the better-studied *Soncus yellow net nucleorhabdovirus*). There is genetic evidence of the Sc4 involvement in virus cell-to-cell movement, and predicted secondary structure arrangement in this protein is dominated by beta-strands, similarly to the 30K-family proteins (39). We could not detect sequence similarity between rhabdovirus MPs and 30K MPs, but HHPred scan showed a specific match between the model for Sc4 and CPs of small icosahedral RNA viruses of insects and plants, with the highest similarity to the model of nodavirus CP (PDB 1NWW; *E*-value < 10^−5^; File S1), suggesting the jelly-roll fold in this putative movement protein as well. The central portion of *Dichoravirus* gp3 also matched a CP in HHPred, in this case the C-terminal domain of turnip yellow mosaic virus CP, PDB 6RTK_C, albeit without statistical significance (File S1). Based on these results, the *Nucleorhabdovirus* and *Dichoravirus* sequences were added to the multiple alignment shown in Figure 1; the asparagine residues aligned with the “D motif” are notable in both cases.

### Evolution of nucleotide and amino acid composition at p19 and p22 nested sequences

**Figure 3:**
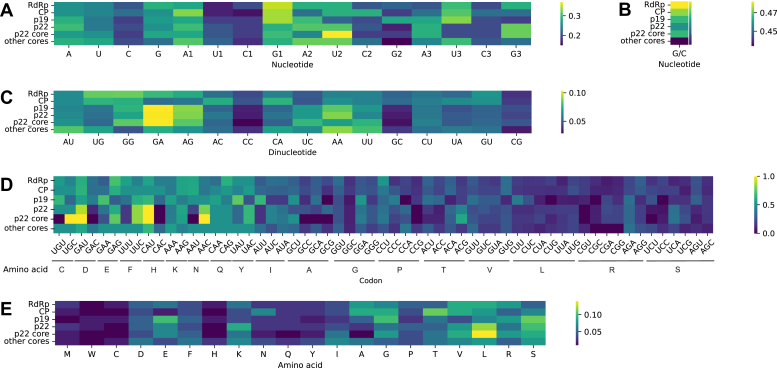
**Composition of nucleotides, dinucleotides, codons, and amino acids in p22 and p19.***A*, nucleotide frequencies of the overprinted genes (in bulk and separately for codon positions 1, 2, 3). *B*, GC content among tombusvirus genes, including the conserved core of the p22 protein. *C*, dinucleotide composition in the overprinted genes. *D*, codon usage in the tombusvirus proteins, in percentages of every codon encoding the same amino acid. *E*, amino acid composition of the tombusvirus proteins. In all heatmaps, the row designations are as follows: RdRp, tombusvirus RNA-dependent RNA polymerase; CP, tombusvirus coat protein; p19, tombusvirus RNA silencing suppressor p19; p22, tombusvirus p22 – entire length; p22 core, p22 region aligned within the 30K family; other cores, homologous regions from the non-overprinted MPs outside the *Tombusvirus* and *Aureovirus* genera.

Unlike most other 30K-family protein genes, the tombusvirus *p22* locus contains a second, nested ORF *p19* in the +2 phase. To understand the mutual constraints imposed on the sequences of these genes and their products, we collected the set of 33 coding-complete tombusvirus genomes and studied the sequence composition of the p22/p19 pairs encoded by this set, as well as in the rest of the genomes and in non-overprinted 30K MPs. The simple sequence statistics for nucleotides (Fig. 3, *A* and *B*), dinucleotides (Fig. 3*C*), codons (Fig. 3*D*), and amino acids (Fig. 3*E*) appear to show relatively few strong differences between the overprinted and non-overprinted parts of virus genomes (remembering, however, that the sample size of tombusvirus overprinted sequences is rather small). For example, the overall CG content in tombusvirus genomes is 48.2%, whereas in the overprinted sequences it is 46.7%; in the non-overprinted homologous regions of other 30K MPs, GC content in 42.4%. Among the dinucleotides, only GA shows excess in tombusvirus p19/p22, while CC and GC are depleted there compared to other tombusvirus genes and to overprinted genes as a whole (Fig. 3*C* and reference (6)).

When codon positions are taken into account, the *p22* sequences have 6% higher frequencies of G and depletion of U by 9% in the first positions compared to a control dataset of 617 non-overprinted MP genes from different viruses (Fig. 3*A*); these shifts, in positions correspond to the second positions in *p19* codons, are not strongly significant (the *p*-values in Mann–Whitney *U* test, respectively, 0.013 and 0.017). The second positions of *p22*, which are the third positions in *p19*, have the frequencies of nucleotides U elevated by more than 30% compared to the base frequency of U across the rest of the genome, and reduced frequencies of the other three bases; in this case, the difference is highly significant, with *p*-value less than 10^−10^. In the third positions of the codons in *p22*, which are the first positions in *p19*, there is somewhat higher frequency of A and depletion of U. Thus, the codon composition of *p19* enriches the protein product for amino acids L, R, S, G and E (encoded by codons starting with nucleotides G and A). There also appears to be a difference in the use of codons with high and low degeneracy - the high-degeneracy codons are used in tombusvirus MPs to the exclusion of low-degeneracy ones (Fig. 3*D*). Specifically, tombusvirus MPs showed higher proportions of four-codon G, L, and six-codon R compared to MPs from other viral families. These observations are in general agreement with the findings by Pavesi *et al.* (12), which, however, have higher statistical power, as they report on a larger aggregated dataset.

**Figure 4:**
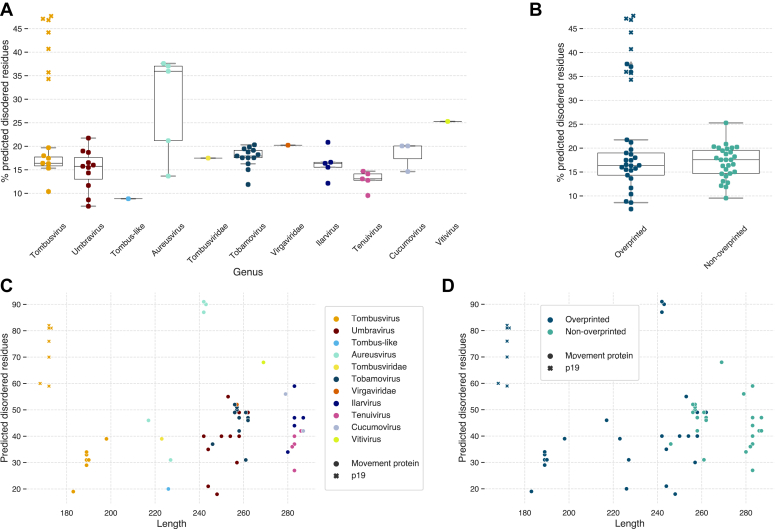
**Protein disorder prediction in MPs and p19.***A*, percentages of amino acids in structurally disordered regions. The *colors* show the taxonomic origin of MPs and p19. p19 values are marked with *crosses*. *B*, percentages of amino acids of overprinted and non-overprinted MPs and p19 located within a predicted structurally disordered region. *C*, amino acids in a predicted structurally disordered region *versus* the length of MPs and p19. The *colors* show the taxonomic origin of MPs and p19. *D*, same as *C*, but the *colors* show whether an ORF us overprinted or not.

**Figure 5:**
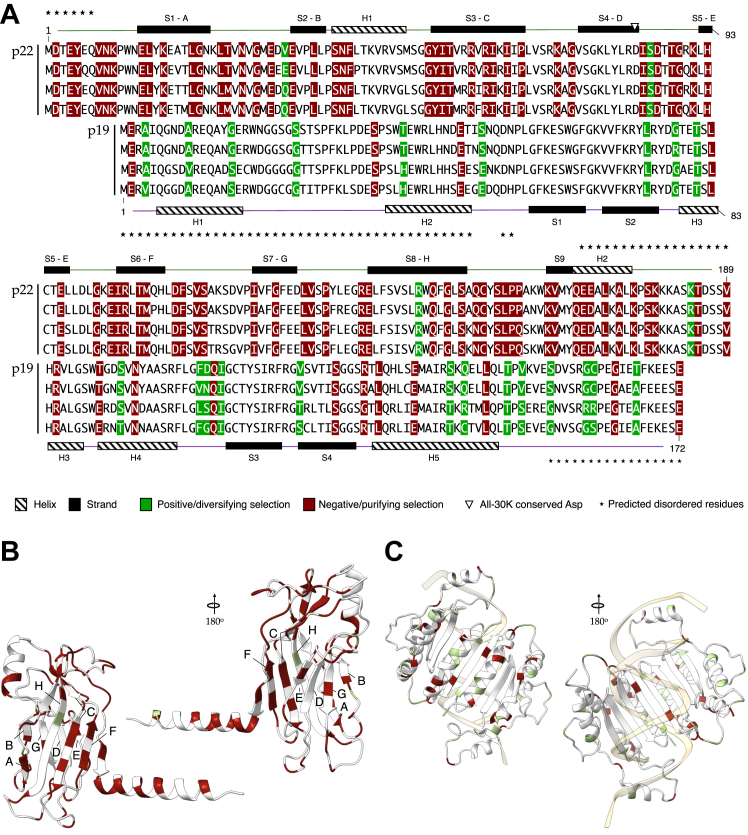
**Selection effects in the evolution of p22 and p19.** Negatively and positively selected sites are marked in *magenta* and *green*, respectively. p22 and p19 sequences are aligned on the basis of the shared dinucleotides in the codons for two aligned amino acids. The predicted protein disorder is shown by *asterisks*. *A*, sites under selection in the amino acid sequence. *B*, selected sites mapped onto the structure of MP. *C*, selected sites mapped onto the structure of p19.

It has been reported that nucleotide and codon biases in the overprinted proteins tend to elevate the frequencies of disorder-promoting amino acids (10). Be it as it may, the predictions of structural disorder in tombusvirus movement proteins indicate lower proportion of amino acids falling within disordered regions than in 30K MPs from other genera (Fig. 4*A* and Table S5). There is no significant difference in this regard between the overprinted and non-overprinted movement proteins, while shorter MPs tend to have fewer disordered residues than the longer ones (Fig. 4, *B*–*D*). On the other hand, p19 has a higher percentage of disorder-promoting amino acids in this dataset, between 30 and 50%, though they tend to cluster towards the termini of the polypeptide chain, outside of the structurally defined alpha+beta region. Note also that the sequence-based algorithm tends to overpredict the disordered regions compared to the known structure of p19 (Fig. 5).

### Selection analyses at the nested coding regions

To understand the selective forces operating upon the two overprinted ORFs in tombusviruses, we generated a maximum-likelihood phylogenetic tree for each of the two nested genes, based on their codon-level nucleotide sequence alignments. We then studied the direction of selection in each codon, using the standard methodology of determining the rates of nonsynonymous substitutions *d*_*N*_ and synonymous substitutions *d*_*S*_ and their per-site *d*_*N*_ − *d*_*S*_ rates difference, using the fixed-effects likelihood (FEL) method. This analysis shows that the protein sequences p22 and p19 are globally under negative and positive selection, respectively (Fig. 5*A*). However, a subset of residues in each protein experience the opposite direction of selection. In both proteins, positively selected residues are found mostly, though not exclusively, in loops, and positively selected residues in p19 are enriched in the regions that mediate the interaction of this protein with dsRNA (Fig. 5*C*).

We classified overlapping codons into four categories based on the direction of selection in each of the two protein sites encoded by the overlap. Fisher’s exact test showed statistically significant enrichment of the codons with positive selection in p19 and negative selection in p22 (*p* = 0.004), rejecting the null hypothesis of independent selection upon each ORF (Fig. S5). Furthermore, in codons that had *d*_*N*_ > *d*_*S*_ in p19 but *d*_*N*_ < *d*_*S*_ in p22, there was a negative correlation between the actual estimated values (*r* = −0.676, 170 df, *p* < 0.0001), which suggests that sites under stronger directional selection in p19 are also under stronger purifying selection in p22. This result is perhaps best understood by noting that a single substitution in a third position of a *p22* codon typically would be synonymous, but would bring with it a single substitution in the first position of the corresponding codon of p19, which would certainly be nonsynonymous. Thus, substitutions in a the third codon positions in a conserved parent ORF (*p22*) have only minimal effect on the parent protein sequence, while facilitating sequence changes in *p19*, and providing variation of the overprinted child ORF, upon which positive selection may act.

We also used the adaptive branch-site random effects likelihood (aBSREL) method to estimate the *ω* = *d*_*N*_/*d*_*S*_ rates ratio per branch for p19 and p22, taking the RNA-directed RNA polymerase (RdRp) genes of the same viruses as a reference. For each branch, estimated *ω*_*p19*_ and *ω*_*p22*_ values were normalized by the corresponding *ω*_*RdRp*_ values, so all branches were brought to a common relative scale. Fig. S5*B* shows that, on average, normalized $\omega_{p19}$ were significantly larger than normalized $\omega_{p22}$ values (paired samples Wilcoxon test *p* < 0.0001), confirming that p19 has evolved under significant directional selection, while p22 has been under significant purifying selection. This is consistent with the previous work on p22/p19 that used a smaller set of viruses (39).

We were also interested in the constraints on the location of the secondary structure elements along the chains of p22 and p19. Analysis of a contingency table of 3 × 3 states (helix, strand, loop) suggests that: codons for the amino acids that are in loops in p22 tend to overlap with the codons that encode strands in p19; regions that encode strands within p22 more commonly encode helices in p19; and the helical regions in p22 overlap loops in p19 at the frequency twice as high as would be expected with the random distribution of the secondary structure elements (3 × 3 Fisher exact tests +10 and +11: *p* < 0.0001; Table S6). Thus, the sequences coding for relatively unstructured loops in the parent gene are compatible with the encoding of strands in the overprinted child protein, and, perhaps more interestingly, the elongated/strand structure, dominant in p22, is compatible with the helical structure in p19 under the “frameshift code”.

Finally, we computed the *d*_*N*_ – *d*_*S*_ values for overlapping codons coding for amino acids located within helices, strands, or loops on each protein (3 × 3 combinations) and tested whether certain combinations of *d*_*N*_ – *d*_*S*_ values were enriched or depleted in each case. The results are presented in Tables S7 and S8. The main conclusion from the inspection of the Tables is that the aforementioned general trend, that is, the predominantly negative selection in the sequence of parent protein p22 and predominantly positive selection in p19, is realized differently depending on which secondary structure elements a particular site encodes. For example, the codons that are under negative selection in p22 gene and encode the amino acids that fall into beta-strands in p22 tends to be under positive selection in p19, but this trend is more evident if in p19 the encoded amino acids fall within helices or loops (Table S7). In another example, sites that are found within loops in p22 are under negative selection if the encoded amino acids are within helices in the p19 structure, but under positive selection if the encoded amino acids in p19 fall into strands or loops (Table S8).

## Discussion

The similarity between the predicted spatial structure of plant virus-specific 30K movement protein family and the ancient capsid module, which is widely distributed among the DNA and RNA viruses infecting all domains of life, has been first suggested based on marginal sequence similarity (24) and recently demonstrated by AlphaFold modeling ((18, 19) and this study). This continues a general theme of plant viruses recruiting copies of CPs to facilitate genome trafficking within and between cells (24). The likely origin of the proto-30K MP in the viruses of the ancestral multicellular freshwater algae and the subsequent evolutionary history with multiple horizontal gene transfer events have been inferred in some detail by Butković *et al.* (19).

Despite the improved understanding of the evolutionary origin of the 30K MP family, the structural models do not provide direct insights into the molecular function of 30K proteins and their role in virus cell-to-cell movement. CPs and MPs play different roles in the course of the infection, with only some potential functional similarities between the two classes of proteins. CPs interact with virus genomic nucleic acids and form oligomers that are incorporated in the higher-order virion structures *e.g*., (40, 41, 42, 43, 44, 45). Similarly, MPs have been long thought to interact with virus genomic nucleic acids (46, 47, 48, 49) and to form higher-order intracellular structures (50, 51, 52, 53, 54, 55, 56, 57). The sites responsible for nucleic acid binding in the CPs, however, are located outside of the main beta-sandwich region (*e.g*., (58, 59, 60)), and mapping of such regions in MPs suggests that neither their sequences nor locations within the protein are evolutionarily conserved (61, 62). Unlike most CPs, the 30K MPs are not known to assemble into oligomers, and the multimeric forms of MPs observed *in vitro* and *in vivo* are not capsid-like (50, 51, 52, 53, 54, 55, 56, 57).

That said, sequence and structural similarity between a single jelly roll and 30K MPs may suggest a potential role for the “D motif” in the MPs. Many icosahedral virions contain divalent cations in stoichiometric amounts, and structural studies show that treating some such virions with EDTA causes particle swelling and destabilization, suggesting that Ca^++^ cations may be required for virion integrity (36, 37, 38). A density suggestive of a metal ion has been detected in the X-ray structures of many virus particles at the interface between three copies of a capsid monomer, and the residues interacting with the cation have been identified in several viruses, including a dyad of aspartic acid residues in the DxxD arrangement in one subunit, as well as additional polar residues in two other subunits within a trimer (36, 37, 38). The first of the two D residues is located at the end of strand E in single jelly-roll capsids, in the position that is directly aligned and superimposed on the D motif in the 30K MP family (Figs. 1 and 2). Interestingly, the entire DxxD signature is found in the 30K MPs from genera *Tombusvirus*, *Aureovirus* and *Cucumovirus*, and variations on this theme are seen in other MPs (Fig. 1), as well as in CPs (Fig. 2). It seems worthwhile to study the role of divalent cations in the stabilization of 30K proteins and in various aspects of their function.

In this work, we also examined another aspect of a 30K MP evolution, *i.e*., the possible structural constraints on the overprinting of *Tombusvirus* MP gene *p22* by a novel, lineage-specific gene *p19*. To be retained in a population, a novel overprinted gene must meet several evolutionary challenges. First, a protein product of sufficient length must be generated. The rate of birth of a random protein-coding sequence by overprinting may be estimated from the first principles, knowing the base composition of the source sequence and the length of the protein product (8, 9, 63, 64, 65, 66). It has been suggested that the compositional skew in the nucleotide sequence and in the encoded amino acid sequence may correlate with the emergence of novel nested ORFs; enrichment of structurally disordered regions in some of the overprinted ORFs has also been seen (13, 66, 67).

The asymmetric selection in the two overprinted genes, predominantly negative in the evolutionarily older, parent gene and predominantly positive in the child gene has been noted before (4, 5, 6, 7, 8). We have run several statistical tests that reveal the underlying complexity of this general evolutionary trend, namely, that at the site level, the sign and magnitude of the asymmetry depend on the identity of the secondary structure element in which the site is located in both ORFs. Be it as it may, the degeneracy of genetic code, as well as the ability of many different sequences to fold into the same tertiary structure, appear to provide for the co-existence of two globular, ordered proteins with different folds and different kinds of selection pressures, encoded by the same polynucleotide in two alternative frames.

As with any other encoded molecule, the product of the overprinted child gene must confer a selective advantage to the organism. In a species with high effective population sizes typical of viruses, such a gene may spread fast enough even if the advantage is small, but, by the same logic, a modest disadvantage of having an overprinted gene (for example, the energetic costs of translating an extra protein) may result in its rapid inactivation and purge from the population, unless the product has a useful function (8). Without a doubt, the retention of an overprinted gene would be facilitated if the child ORF could grow in length in a stepwise fashion, where each of the intermediate-length products was itself adaptive. In fact, such a scenario of p19 evolution may be tentatively proposed.

The spatial structure of p19 is a specific permutation of the RAGNYA fold, found in diverse DNA-binding and RNA-binding domains (16); the overall fold consists of a four-stranded beta-sheet with two or more helices arranged on the convex side of the sheet, and the polynucleotide is bound by the opposite, concave side of the sheet (15, 16). In p19, the arrangement of helices (H) and strands (S) along the sequence is N-HSSHHSSH-C, and to bind RNA, the protein forms a dimer with the combined eight-strand beta-sheet. The function of p19 involves binding and sequestering short double-strand RNA, which is presumably how p19 counters the host RNAi-based anti-virus systems (15). Notably, half of all contacts between p19 and the RNA is located within the two adjoining strands three and 4 (Fig. 5*C*).

We speculate that an ancestor of p19 consisting of 20 to 25 amino acids spuriously emerged by recoding of a segment within *p22*. The encoded peptide may have folded into a simple beta-hairpin, perhaps with an additional coil-like element stabilizing the back surface; such a primitive entity, the ancestor of strands 3 and 4, already would have been able to bind RNA, providing a rudimentary anti-RNA-interference function. A C-terminal extension of such an ORF coding a helix could have occurred next; this would have a potential of facilitating the protein dimer formation, much like in p19, where the intersubunit interactions in dimers are mostly mediated by the strand 4 and the C-terminal helix. The dimer formation would allow for an interaction with a double-strand polynucleotide. The next extension of the *p19* ORF, this time towards the N-terminus, could have added a strand 2, or an entire second beta-hairpin of strands 1 and 2, enlarging the sheet and adding several protein-RNA contacts. Finally, additional helices and specific conserved residues within them may have evolved, which would help stacking of some side chains with the nucleobases and target size selection observed in the present-day p19 homologs (15). This scenario of stepwise addition of structural elements to the conserved core of the fold appears to be partially supported by the analysis of the aureovirus p14, a sequence homolog of p19 that is also encoded by a gene nested within the movement protein ORF. The p14 protein is known to bind double-strand RNAs but does not discriminate targets by size (68), and the smaller p14 appears to lack the equivalent of p19 N-terminal sequence and structure elements that are mainly responsible for size selection (see Fig. S6).

The comparison of p19 to the structure databases with the Dali program shows a structural similarity between p19 and the C-terminal DNA-binding domain of the Y-family DNA polymerases, another RAGNYA-fold domain, with the *z*-scores below statistical significance (3 or lower). At such a significance level, and in the absence of sequence-level similarity signal, it is notoriously hard to determine whether the sequences (*i*) are homologous but have diverged beyond recognition, retaining only the structural similarity (“structures are more conserved in [divergent] evolution than sequences”) or (*ii*) are not homologous and have evolved towards similar structures from spatially different folds, in a truly convergent fashion. The examples of *bona fide* structural convergence towards the same fold noted in the literature are extremely rare and are limited to engineered proteins, rationally designed to share a fold with a known protein in the absence of sequence similarity, or to chimeric “folds” purposely combined *in silico* from sequence-adjoining segments of two distinct evolutionarily conserved domains (69, 70). The evolutionarily recent, tombusvirus-specific sequence of p19, which clearly does not share a common ancestor with the widespread Y-family DNA polymerases or with other RAGNYA-fold proteins, thus provides a startling example of convergent evolution of protein folds in nature.

## Experimental procedures

### Datasets and alignments

The dataset of *Tombusvirus* p19 and p22 protein homologs was collected from the NCBI NR protein sequence database; it contains 36 pairs of sequences, of which 33 belong to coding-complete virus genomes (File S2). The dataset of the 30K superfamily of plant virus MPs was constructed as follows. First, seven families were identified in the PFAM database that include the sequences known to be homologous to the 30K proteins (Table S1). The set of the 30K homologs is informally referred to as “family” or “superfamily” in this work, even though PFAM splits it into several PFAM families, and the cluster structure of the entire dataset appears to be complex.

The NCBI nonredundant protein database (NR) was used as the search space, and scans with individual protein sequences were done using the PSI-BLAST program (71) with *E*-value = 0.001, and with the profile models using the HMMER program (72). The union of all significant virus matches was collected and filtered by a minimum percentage of sequence identity of 30% and a minimum alignment length of 100 amino acids.

The dataset of MPs was submitted to the CLANS server (73) using default configuration with a threshold *E*-value = 0.0001, in order to identify and visualize relationships in an all-*versus*-all cluster map (Fig. S2). The method of convex clustering in CLANS was employed to group the sequences together, with a standard deviation cutoff of 0.1 and the minimum of sequences per cluster was set to 30. Afterwards, the Euclidean distance in a 3-dimensional space was calculated and represented using a custom Python code and used as measure of dissimilarity between the cluster containing *Tombusvirus* MP sequences and the rest of groups. *Tombusvirus* MPs are among the shortest in the 30K family, therefore other members of the family were trimmed to retain only the regions that could be aligned to the *Tombusvirus* p22 with MAFFT (74). Each successive cumulative alignment was manually refined using UGENE (75) on the basis of the secondary structures predicted with PSIPRED (76).

### AlphaFold *ab initio* folding, model refinement, and evaluation

The structure of the conserved cores of the 30K proteins was predicted AlphaFold2 (17) implemented in the ColabFold notebooks (77) running on Google Collaboratory (https://colab.research.google.com/github/sokrypton/ColabFold/blob/main/AlphaFold2.ipynb). We used the default settings with Amber relaxation, providing the alignment described above and the following configuration parameters: template_mode = none, msa_method = custom (or = mmseqs2_uniref_env, if the resulting predicted local distance difference test (pLDDT) was greater than 80 (78)), pair_mode = paired, model_type = AlphaFold2-ptm, rank_by = pLDDT, num_models = 5, max_recycles = 3. The custom MSA contained 684 different species. A total of 36 MPs sequences from tombusvirus species were individually submitted as queries, giving very similar results (not shown). Additionally, we also built models of movement proteins from other 27 genera (Table S2).

The following analysis is anchored on the full sequence and structure of the movement protein from the tomato bushy stunt virus (*e.g*., QYA72475.1). The improvement of the local stereochemistry of the top-ranked protein structure model was made with locPREFMD (79).

### Structural comparisons

The shared region structures of the different families of MPs were superimposed by Matchmaker (ChimeraX tool (80)), using Smith-Waterman and Needleman-Wunsch alignment algorithms. A secondary structure term was included in the superimposition score, with a weight of 0.7 (hence, the weight of residue similarity was 0.3). The rest of the parameters were set to default values. The resulting RMSD values were used as the measure of structural similarity. Hierarchical clustering was performed in Python (Scipy.cluster.hierarchy module) with Needleman-Wunsch distances and using the centroid clustering method, as this method returned the highest cophenetic correlation coefficient. We used the DALI Server (81) to compare the refined structure of the *Tomato bushy stunt virus* MP against previously known protein structures in the PDB database.

### Comparative analysis of overprinted and non-overprinted genes

In-house Python code was written to calculate nucleotide, dinucleotide, codon, and amino acid composition of genes and proteins. Codon usage was calculated in terms of the percentage of every codon encoding the same amino acid. Comparisons were made between the conserved core of p22 (overprinted sequence) *versus* the ORFs for the RdRp, the CP, p22 and p19 and other movement protein cores. For that purpose, the previously built thick alignment of movement proteins was used as a reference. We confirmed the significance of composition differences with the Wilcoxon test for paired data and the Mann-Whitney *U* test for the case of unpaired ones.

### Selection analysis of tombusvirus overprinted genes

A total of 36 paired sequences of p19 and p22 genes and their corresponding genomes were available in the GenBank database. For the alignments of the coding sequences of p19 and p22, their protein sequences were aligned with the ClustalX algorithm (82) as implemented in MEGA X (83), and then reverse-translated to the corresponding DNA multiple alignments based on the open reading frames with RevTrans (84). JModelTest (85) was used for statistical selection of the best-fit model of nucleotide substitution, under either Akaike or Bayes information criteria (AIC and BIC, respectively). The maximum likelihood phylogenies were generated with a bootstrap value of 1000 and under its best model with IQ-TREE (86) and rendered using iTOL (87) and the R package phytools 1.5.1 (88). Alignments and phylogenies of *p19* and *p22* genes were given as input for the selection analyses performed locally with HYPHY 2.5.32 (89) with fixed effects likelihood (FEL) algorithm. The program analyzes the rates of nonsynonymous substitutions *d*_*N*_ and synonymous substitutions *d*_*S*_ and computes the *d*_*N*_ − *d*_*S*_ difference at each site. For their comparison, 10 amino acids/codons of p22 after the start codon (+10) were considered as pairing data for each one of p19. Fisher’s exact tests were performed to detect dependencies between p19 and p22 substitution rates. We also calculated Pearson correlation coefficients to measure associations between the strength of selection at both ORFs. Contingency tables 3 × 3 were calculated with the overlap +10 and +11 between p19 and p22 secondary structures to search for structural patterns of correlated selection between these two proteins. The tables contained negative, neutral and positive selection counts for both sequences, and considered all amino acids, alpha-helices, beta-strands or loops separately. Freeman-Halton extension of the Fisher exact probability test was used when the size of data was lower than 90 using the VassarStats server (http://vassarstats.net/fisher3x3.html).

Further examination was carried out by running selection analyses with adaptive branch-site random effects likelihood (aBSREL) (90) as implemented in HYPHY 2.5.32. For comparing *ω* ratios among coding sequences, we used a concatenation of the alignments of *p19*, *p22*, and *RdRp* ORFs from tombusviruses, from which the species tree was estimated. To make *ω* values comparable among viral lineages that might show overall differences in rates of evolution, values for *p19* and *p22* were scaled by the corresponding estimates obtained for the *RdRp*: $\omega_{x}=\omega_{x}/\omega_{RdRp}$ with *x* ∈ {*p19*, *p22*}. For visualization, $\omega_{x}$ data were asinh-transformed. Comparisons between per branches *ω*∗ values for each ORF were done using the Wilcoxon paired-samples test. Additionally, we employed a one-sample *t* test to test for significant departures from the null hypothesis of no selection.

### Structural disorder prediction

The sequences of 31 non-overprinted movement proteins, 25 overprinted ones, and seven sequences of p19 were included for a comparative analysis of their composition of disordered amino acids. The prediction was carried out using the webserver flDPnn (http://biomine.cs.vcu.edu/servers/flDPnn/).

## Data availability

All code written for this paper and the supporting data have been deposited at https://git.csic.es/sfelenalab/nested-genes-viruses.

## Supporting information

This article contains supporting information.

## Conflict of interest

The authors declare that they have no known competing financial interests or personal relationships that could have appeared to influence the work reported in this paper.
