# Supplementary figures and images for: Emergence of two distinct spatial folds in a pair of plant virus proteins encoded by nested genes
Source: J Biol Chem. 2024 Mar 24;300(5):107218. doi: 10.1016/j.jbc.2024.107218 (PMC11044054; doi:10.1016/j.jbc.2024.107218)

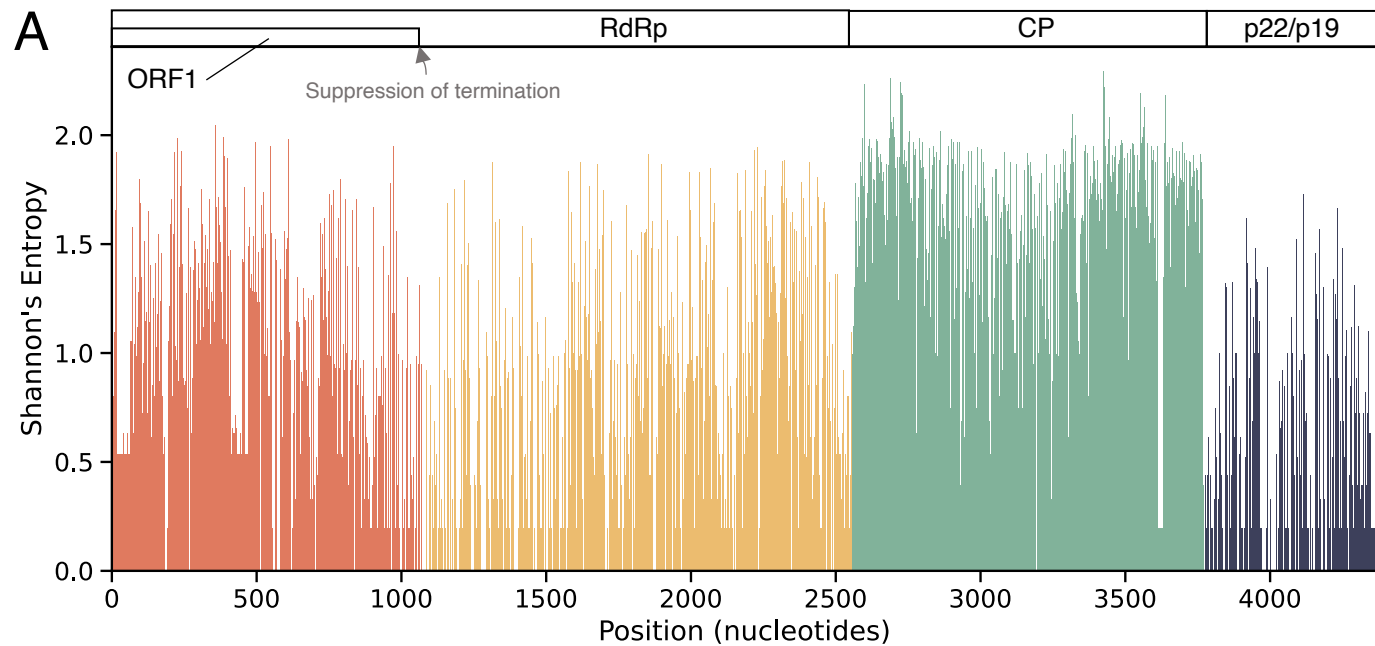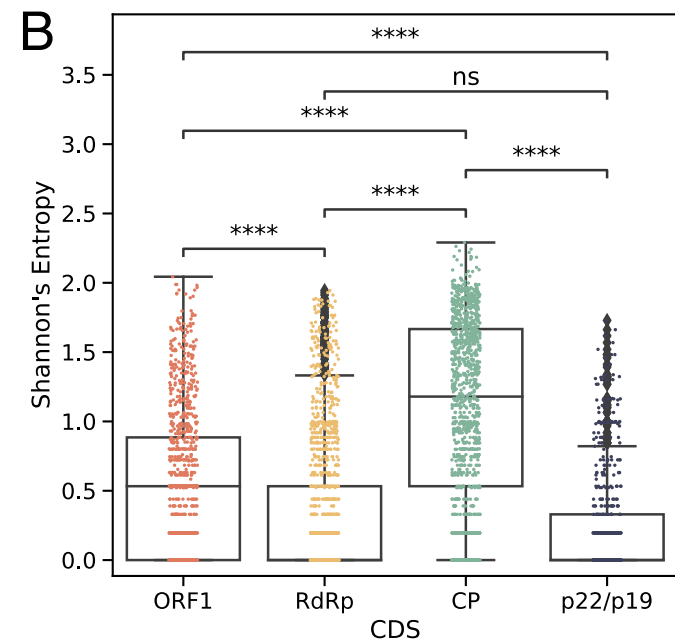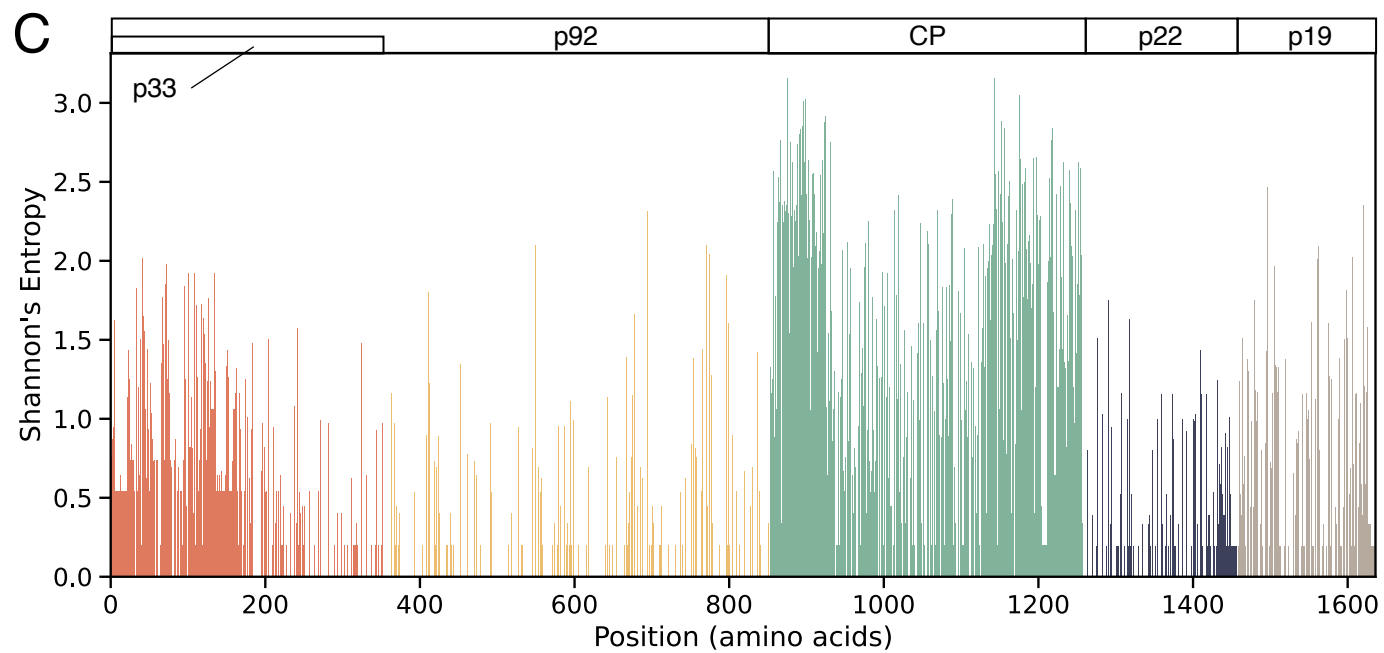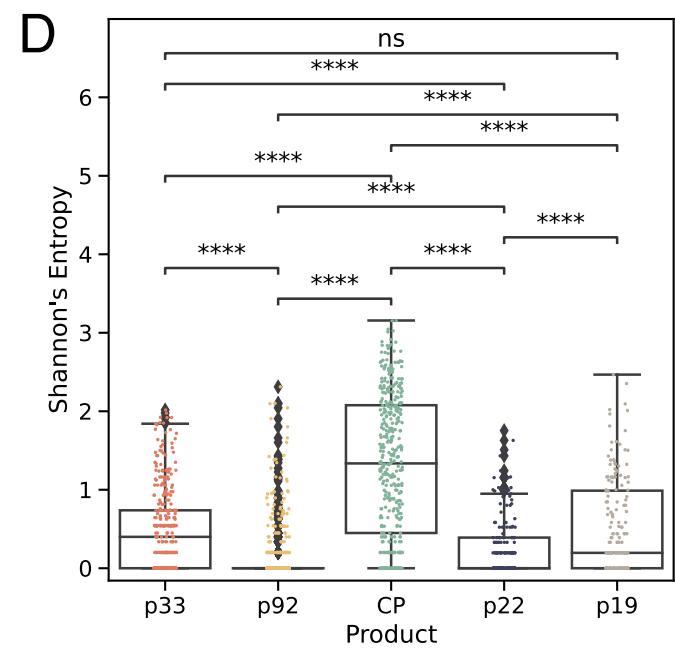

Supplement: Supporting Figure S1 [file mmc1.pdf]

A

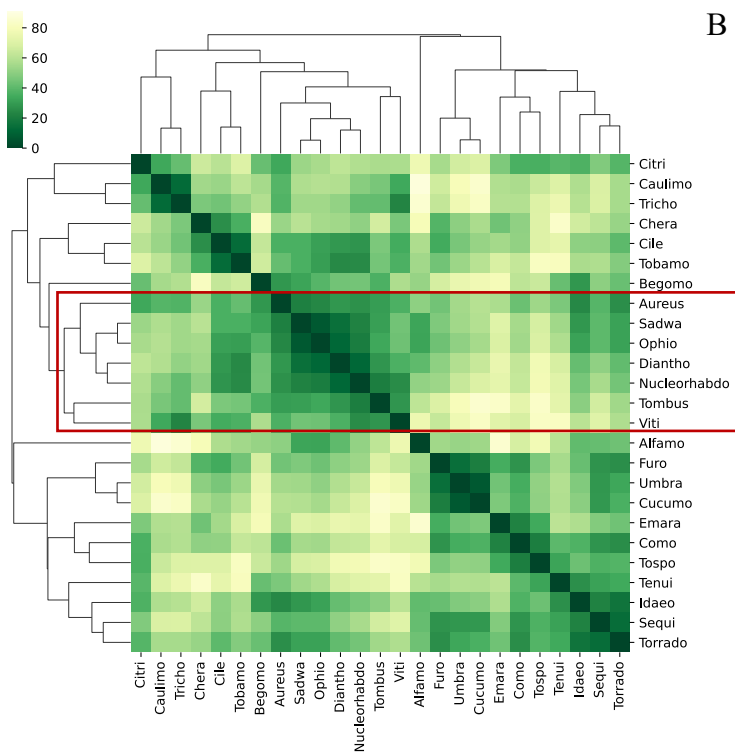

B

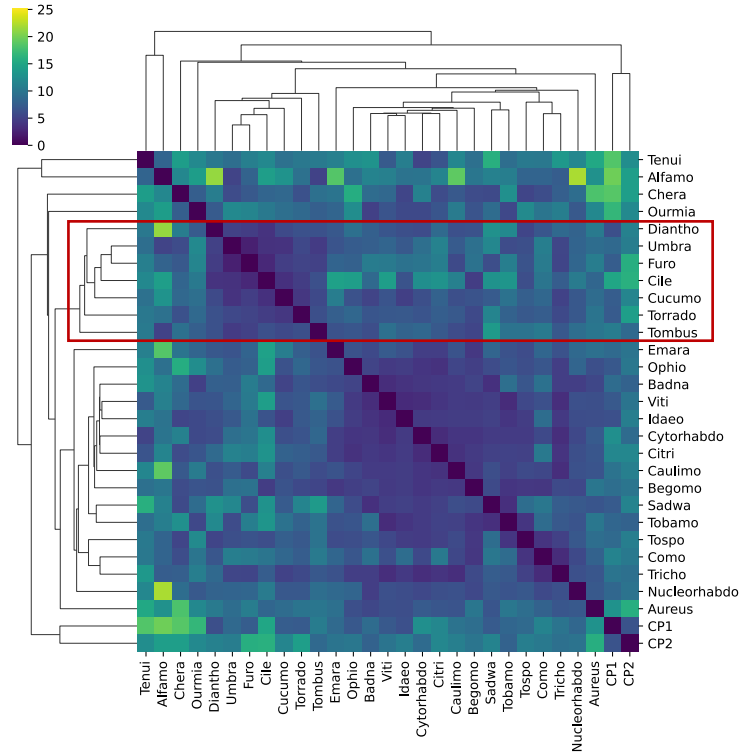

Supplement: Supporting Figure S2 [file mmc2.pdf]

**A**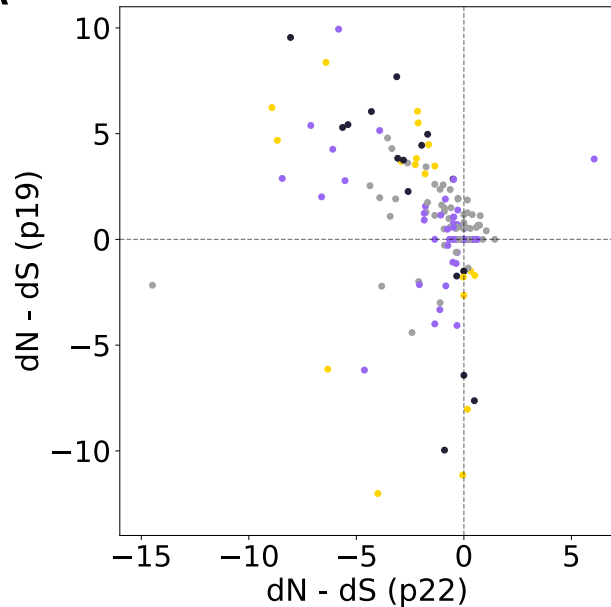**B**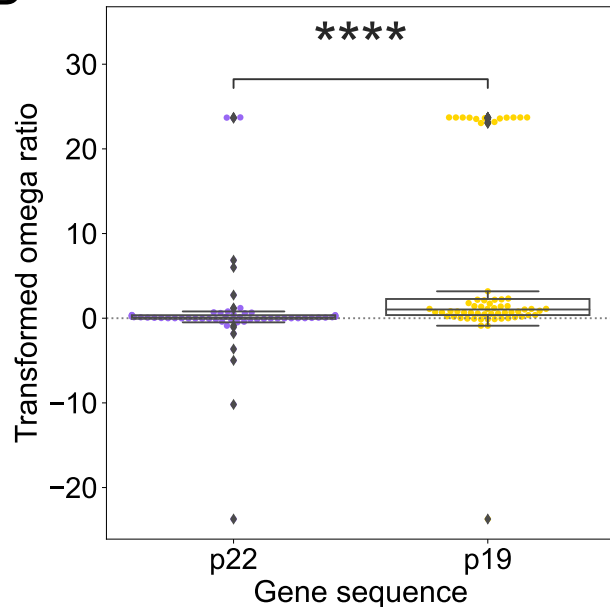

Supplement: Supporting Figure S5 [file mmc5.pdf]

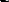 Helix    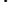 Strand

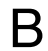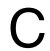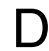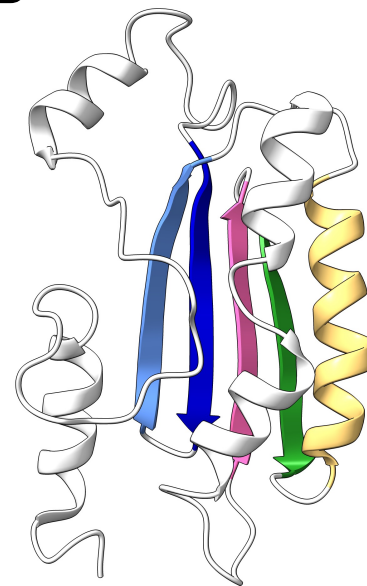

Supplement: Supporting Figure S6 [file mmc6.pdf]
