# Supplementary material for: Emergence of two distinct spatial folds in a pair of plant virus proteins encoded by nested genes
Source: J Biol Chem. 2024 Mar 24;300(5):107218. doi: 10.1016/j.jbc.2024.107218 (PMC11044054; doi:10.1016/j.jbc.2024.107218)
Supplement: Supporting Figure S3 [file mmc3.pdf]

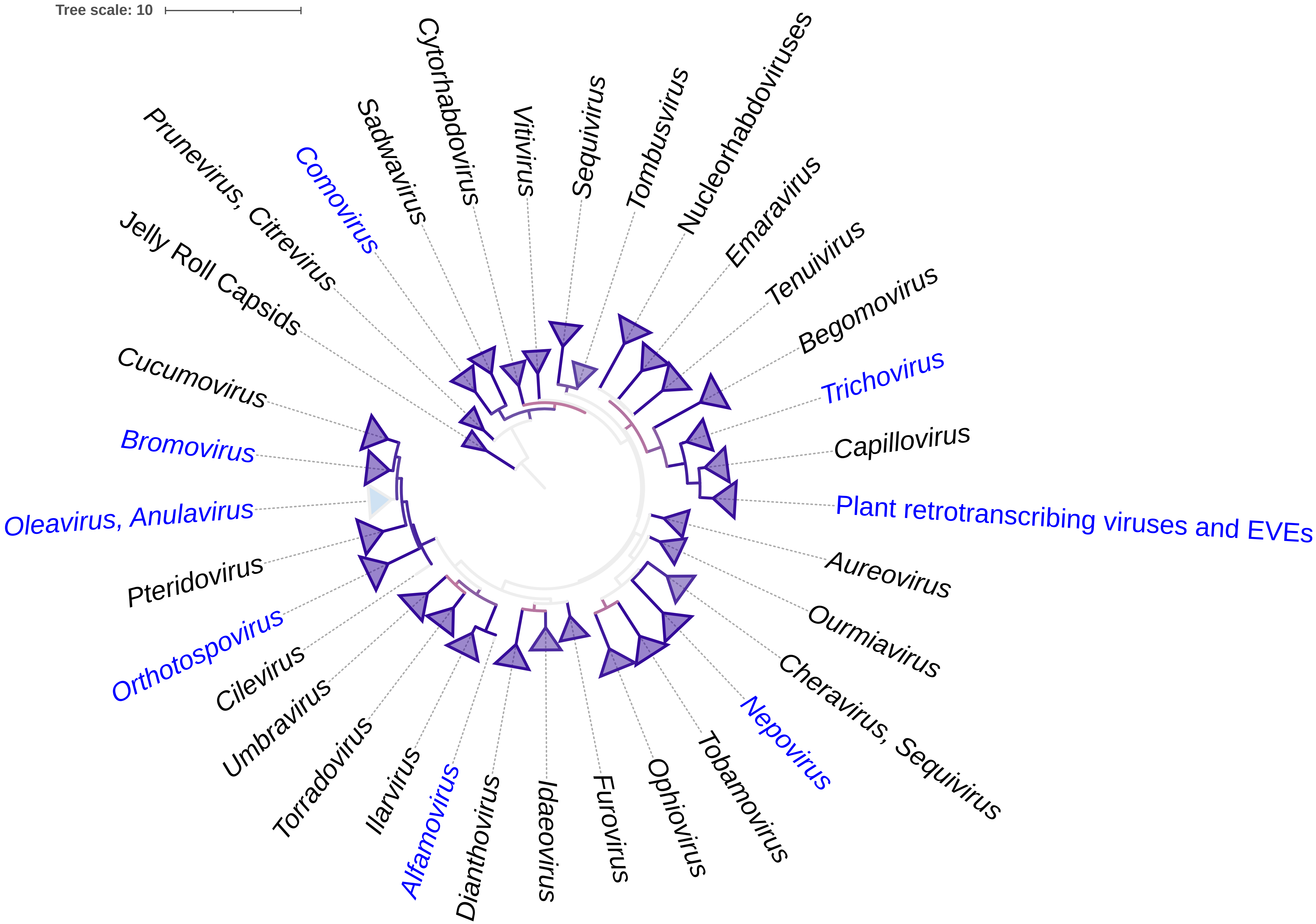
Tree scale: 10
Cytorhabdovirus
Sadwavirus
Vitivirus
Sequivirus
Tombusvirus
Nucleorhabdoviruses
Comovirus
Prunevirus, Citrevirus
Emaravirus
Jelly Roll Capsids
Tenuivirus
Begomovirus
Cucumovirus
Trichovirus
Bromovirus
Capillovirus
Oleavirus, Anulavirus
Plant retrotranscribing viruses and EVEs
Pteridovirus
Aureovirus
Orthotospovirus
Ourmiavirus
Cilevirus
Umbravirus
Cheravirus, Sequivirus
Torradovirus
Nepovirus
Ilarvirus
Tobamovirus
Alfamovirus
Ophiovirus
Dianthovirus
Furovirus
Idaeovirus

Viruses

+-Shotokuvirae
I \-Cressdnaviricota
I   \-Repensiviricetes
I   \-Geplafuvirales
I   \-Geminiviridae
         \-**Begomovirus**
          \-Cabbage leaf curl virus

+-Orthornavirae
I +-Pisuviricota
I I \-Pisoniviricetes
I I   \-Picornavirales
I I   \-Secoviridae
I I   +-**Torradovirus**
I I   I \-Torradovirus lycopersici
I I   I   \-Tomato torrado virus
I I   +-**Cheravirus**
I I   I \-Cheravirus avii
I I   I   \-Cherry rasp leaf virus
I I   +-**Sadwavirus**
I I   I \-Sadwavirus citri
I I   I   \-Satsuma dwarf virus
I I   +-**Sequivirus**
I I   I \-Sequivirus pastinacae
I I   I   \-Parsnip yellow fleck virus
I I   \-**Comovirus**
I I   \-Comovirus vignae
I I   \-Cowpea mosaic virus
I +-Kitrinoviricota
I I +-Alsuviricetes
I I I +-Martellivirales
I I I I +-Kitaviridae
I I I I I \-**Cilevirus**
I I I I I   \-Cilevirus leprosis
I I I I I   \-Citrus leprosis virus C
I I I I +-Mayoviridae
I I I I I \-**Idaeovirus**
I I I I I   \-Idaeovirus rubi
I I I I I   \-Raspberry bushy dwarf virus
I I I I +-Bromoviridae
I I I I I +-**Alfamovirus**
I I I I I I \-Alfalfa mosaic virus
I I I I I \-**Cucumovirus**
I I I I I   \-Cucumber mosaic virus
I I I I \-Virgaviridae
I I I I   +-**Furovirus**
I I I I   I \-Soil-borne wheat mosaic virus
I I I I   \-**Tobamovirus**
I I I I   \-Tobacco mosaic virus
I I I \-Tymovirales
I I I   \-Betaflexiviridae
I I I   +-**Vitivirus**

```
| | |   | \-Actinidia virus B
| | |   +-Citrivirus
| | |   | \-Citrus leaf blotch virus
| | |   \-Trichovirus
| | |     \-Apple chlorotic leaf spot virus
| | \-Tolucaviricetes
| |   \-Tolivirales
| |     \-Tombusviridae
| |       +-Umbravirus
| |       | \-Umbravirus maculacarotae
| |       |   \-Carrot mottle virus
| |       +-Aureusvirus
| |       | \-Aureusvirus aurei
| |       |   \-Pothos latent virus
| |       +-Dianthovirus
| |       | \-Dianthovirus trifolii
| |       |   \-Red clover necrotic mosaic virus
| |       \-Tombusvirus
| |         \-Tombusvirus lycopersici
| |           \-Tomato bushy stunt virus
| \-Negarnaviricota
|   +-Ellioviricetes
|   | \-Bunyavirales
|   |   +-Phenuiviridae
|   |   | \-Tenuivirus
|   |   |   \-Tenuivirus oryzaclavatae
|   |   +-Tospoviridae
|   |   | \-Orthotospovirus
|   |   |   \-Orthotospovirus tomatomaculae
|   |   \-Fimoviridae
|   |     \-Emaravirus
|   |       \-Emaravirus toordali
|   +-Milneviricetes
|   | \-Serpentovirales
|   |   \-Aspiviridae
|   |     \-Ophiovirus
|   |       \-Ophiovirus citri
|   |         \-Citrus psorosis virus
|   \-Monjiviricetes
|     \-Mononegavirales
|       \-Rhabdoviridae
|         \-Betanucleorhabdovirus
|           \-Betanucleorhabdovirus retesonchi
|             \-Sonchus yellow net nucleorhabdovirus
\-Pararnavirae
  \-Artverviricota
    \-Revtraviricetes
      \-Ortervirales
        \-Caulimoviridae
          \-Caulimovirus
            \-Caulimovirus tessellobrassicae
              \-Cauliflower mosaic virus
```
