# Supplementary material for: Emergence of two distinct spatial folds in a pair of plant virus proteins encoded by nested genes
Source: J Biol Chem. 2024 Mar 24;300(5):107218. doi: 10.1016/j.jbc.2024.107218 (PMC11044054; doi:10.1016/j.jbc.2024.107218)
Supplement: Supporting Figure S4 [file mmc4.pdf]

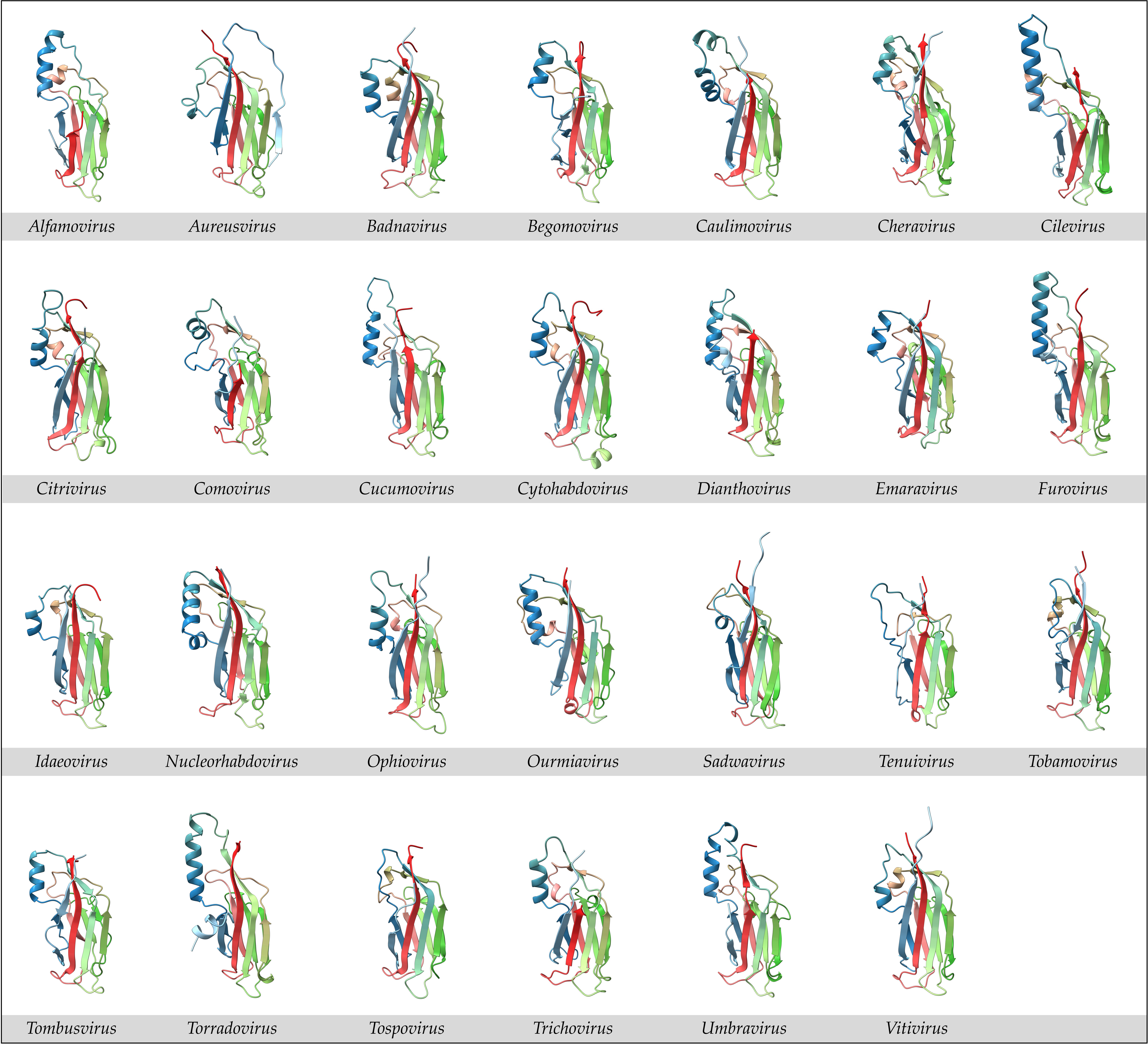
Alfamovirus
Aureusvirus
Badnavirus
Begomovirus
Caulimovirus
Cheravirus
Cilevirus
Citrivirus
Comovirus
Cucumovirus
Cytohabdovirus
Dianthovirus
Emaravirus
Furovirus
Idaeovirus
Nucleorhabdovirus
Ophiovirus
Ourmiavirus
Sadwavirus
Tenuivirus
Tobamovirus
Tombusvirus
Torradovirus
Tospovirus
Trichovirus
Umbravirus
Vitivirus
