# Supplementary material for: Emergence of two distinct spatial folds in a pair of plant virus proteins encoded by nested genes
Source: J Biol Chem. 2024 Mar 24;300(5):107218. doi: 10.1016/j.jbc.2024.107218 (PMC11044054; doi:10.1016/j.jbc.2024.107218)
Supplement: Supporting Information [file mmc7.docx]

**Supporting Figure S1. Sequence conservation in tombusvirus genes.** A) Nucleotide-level Shannon entropy along the tombusvirus genome alignment. B) Amino acid-level Shannon entropy in concatenated protein alignment of tombusvirus proteins. C) Distribution of the nucleotide-level Shannon entropy values for each of tombusvirus genes. D) Distribution of the amino acid-level Shannon entropy values for each of tombusvirus proteins. In C and D, horizontal brackets indicate pairwise comparisons, and for each pair, the p-value in the Mann-Whitney U rank test is indicated as follows: ns, not significant (p-value between 0.05 and 1); *, 0.01 < p ≤ 0.05; **, 0.001 < p ≤ 0.01; ***, 0.0001 < p ≤ 0.001; ****, p < 0.0001 .

**Supporting Figure S2.** Clustering analysis of sequence and structure similarities between 30K-family movement proteins.

A. Cluster 30K proteins' by sequence similarity computed by the CLANS server. Clusters are annotated by the truncated genus name, except the following: genera *Anulavirus*, *Bromovirus* and *Cucumovirus* are included in the Cucumo cluster; *Capillovirus*, *Cytorhabdovirus and Trichovirus* are included in the Tricho cluster; *Cheravirus* and *Nepovirus* are in the Chera cluster; *Alfamovirus* and *Ilarvirus* are in the Alfamo cluster.

B. Structural similarity (RMSD distances) between 30K MPs and selected jelly-roll capsid proteins. CP1, PDB 6SCL subunit B; CP2, PDB 4LLF subunit A.

**Supporting Figure S3.** Phylogeny and taxonomic distribution of 30K MP proteins.

A. Stylized maximum-likelihood phylogenetic tree of 30K MPs, obtained with the PhyML approach with bootstrap-by-transfer resampling. Tree is rooted on the jelly-roll capsid protein outgroup, clades are collapsed mostly at the genus level, and the clades with support of more than 70 percent are indicated by the shades of purple.

B. Schematic of the plant virus taxonomy maintained by NCBI. Only the clades encoding the 30K MP are retained; the representative species including in this analysis are underlined.

**Supporting Figure S4.** Three-dimensional models of 30K movement protein domains from various genera of plant viruses.

**Supporting Figure S5. Supporting Figure S4. Asymmetric distribution of selected codons among overprinted ORFs.**

A. Counts of the sites experiencing directed selection in at least one of the two ORFs.

B. The ω values of p22 and p19 normalized by the corresponding ω values obtained for RdRp and argsh-transformed for visualization. The p-value is < 0.0001.

**Supporting Figure S6.** Model of aureovirus p14 protein, a shorter homolog of p19.

A. Known secondary structure elements in p19 structure (PDB 6BJV) and predicted secondary structure of aureovirus p14 (from AlphaFold prediction, this study). Homologous elements superimposable in sequence are highlighted in the same color.

B. AlphaFold model of p14 color-coded based on pLDDT scores.

C. AlphaFold model of p14 color-coded according to A.

D. Structure of CIRV p19 protein in complex with siRNA (6BJV) color-coded according to A.
