# Supplementary material for: Emergence of two distinct spatial folds in a pair of plant virus proteins encoded by nested genes
Source: J Biol Chem. 2024 Mar 24;300(5):107218. doi: 10.1016/j.jbc.2024.107218 (PMC11044054; doi:10.1016/j.jbc.2024.107218)
Supplement: Supporting File S1 [file mmc8.rtf]

Dichoravirus

Query         Q_4534400_2
Match_columns 277
No_of_seqs    1 out of 3
Neff          1
Searched_HMMs 81620
Date          Sat Jul  1 15:29:55 2023
Command       hhsearch -cpu 8 -i ../results/full.a3m -d /cluster/toolkit/production/databases/hh-suite/NCBI_CD/NCBI_CD -d /cluster/toolkit/production/databases/hh-suite/mmcif70/pdb70 -o ../results/4534400_2.hhr -oa3m ../results/4534400_2.a3m -p 20 -Z 250 -glob -z 1 -b 1 -B 250 -ssm 2 -sc 1 -seq 1 -dbstrlen 10000 -realign -mact 0.0 -maxres 32000 -contxt /cluster/toolkit/production/bioprogs/tools/hh-suite-build-new/data/context_data.crf 

 No Hit                             Prob E-value P-value  Score    SS Cols Query HMM  Template HMM
  1 6RTK_C Major capsid protein; v  27.4      93  0.0011   19.1   8.6  132    1-172    71-202 (202)

No 1
>6RTK_C Major capsid protein; virus;{Turnip yellows virus (isolate FL-1)}
Probab=27.41  E-value=93  Score=19.12  Aligned_cols=132  Identities=18%  Similarity=0.247  Sum_probs=19.6  Template_Neff=6.700

Q ss_pred             CCCCcccceeEEeecchHHHHHHhhhhhCCCCCCCceeeeeeeEeeecCCccCCCceEEEEecCcCCCccccCceeEeee
Q Q_4534400_2       1 VTGSEGEGRITMVRKPILFDIMNSLNSILPTSTKPTWIMGMAIKWIPTCDLSTTGAIKVSIQNKAVNNPVLRDHTVVSMT   80 (277)
Q Consensus         1 vtgsegegritmvrkpilfdimnslnsilptstkptwimgmaikwiptcdlsttgaikvsiqnkavnnpvlrdhtvvsmt   80 (277)
                      +...-|+.--+..-.|-+-+...-|+.|+..-.+- -|..+.|+|+|.|--.+.|+|-+.+.-...... +.+ .+  ++
T Consensus        71 ~~~~~~~s~g~i~f~P~~s~~~~wl~~ia~~y~~Y-ri~~l~i~y~p~~stTt~Gsv~~~~D~d~~~~~-~~s-~i--~~  145 (202)
T 6RTK_C           71 KDNLAGSSSGAITFGPSLSDCPAFSNGMLKAYHEY-KISMVILEFVSEASSQNSGSIAYELDPHCKLNS-LSS-TI--NK  145 (202)
T ss_dssp             ECCCCSSEEEEEEESTTCCSCHHHHHHHHHHCSEE-EEEEEEEEEECCSCSSCCCEEEEEEESSSCCSS-CCC-CC--EE
T ss_pred             ecCCCCccceeEEecCChhhCchhHHHHHHHHhee-EEEEEEEEEEeCCCCCCCceEEEEEcCCCCCCC-cCc-ce--ee
Confidence            00000000000000111000000000000000000 112233333333333333333333322211100 000 00  00


Q ss_pred             cceeCCeEeEeccccccccccCCCCCccceeEeecCccCCCcccccCcEEEEeeecCCCCCceeeecceeeeecceeccc
Q Q_4534400_2      81 QRVTTPFEVQYTSSSKLANRTGTRGNPWMYTYCIEGMDDAPIDMEVGDIVVMPMIRSDDTTTQWYEGVKCNVYGGYFPLN  160 (277)
Q Consensus        81 qrvttpfevqytsssklanrtgtrgnpwmytyciegmddapidmevgdivvmpmirsddtttqwyegvkcnvyggyfpln  160 (277)
                      -.+..+.+..... +.|      .+.+|.++-     +|.      +-|++..    +..+          ...|.|-..
T Consensus       146 ~~v~k~~~l~~~~-~~i------~~~~w~~~s-----~Dq------f~i~~~g----~g~s----------~~~G~~~it  193 (202)
T 6RTK_C          146 FGITKPGKRAFTA-SYI------NGTEWHDVA-----EDQ------FRILYKG----NGSS----------SIAGSFRIT  193 (202)
T ss_dssp             EESSSCEEEEECH-HHH------TCSSCEETT-----SCC------EEEEEEE----ESCS----------SCCCEEEEE
T ss_pred             ccCCCCeEEEECH-hHc------CCCceEEcC-----CCe------EEEEEEe----CCCC----------CCceEEEEE
Confidence            1111111111110 000      112222210     000      0000000    0000          000111111


Q ss_pred             CcEEEEecCChh
Q Q_4534400_2     161 IPVVTYCAPGPR  172 (277)
Q Consensus       161 ipvvtycapgpr  172 (277)
                      .-|...   +|.
T Consensus       194 Y~V~l~---~Pk  202 (202)
T 6RTK_C          194 IKCQFH---NPK  202 (202)
T ss_dssp             EEEEEE---SCC
T ss_pred             EEEEEe---CCC
Confidence            100000   000


Nucleorhabdovirus

Query         Q_5943908_1
Match_columns 192
No_of_seqs    13 out of 15
Neff          3.82437
Searched_HMMs 77898
Date          Mon Oct 10 14:25:32 2022
Command       hhsearch -cpu 8 -i ../results/full.a3m -d /cluster/toolkit/production/databases/hh-suite/mmcif70/pdb70 -d /cluster/toolkit/production/databases/hh-suite/NCBI_CD/NCBI_CD -o ../results/5943908_1.hhr -oa3m ../results/5943908_1.a3m -p 20 -Z 250 -glob -z 1 -b 1 -B 250 -ssm 2 -sc 1 -seq 1 -dbstrlen 10000 -realign -mact 0.0 -maxres 32000 -contxt /cluster/toolkit/production/bioprogs/tools/hh-suite-build-new/data/context_data.crf 

 No Hit                             Prob E-value P-value  Score    SS Cols Query HMM  Template HMM
  1 4NWW_A Capsid protein; beta ba  81.7       4 5.2E-05   26.5  10.4  172    3-192     1-192 (358)
  2 4NWV_A Capsid protein; beta ba  60.8      18 0.00023   23.0  11.7  185    1-192    15-232 (391)
  3 3ZXA_C CAPSID PROTEIN; VIRUS,   56.8      22 0.00028   22.5  10.8  183    1-192    40-263 (347)
  4 6MRM_C Capsid protein; RCNMV,   52.1      27 0.00035   22.0  11.1  183    1-192    17-234 (339)
  5 4Y5Z_Q Immunoglobulin G-bindin  51.6      28 0.00036   21.9  11.9  177    1-181    60-282 (282)
  6 1OPO_B Coat protein; PLANT VIR  50.0      30 0.00039   21.7  13.1  187    1-192    30-269 (348)
  7 1F2N_C CAPSID PROTEIN; PLANT V  33.5      65 0.00084   19.9  11.4  172    1-181    13-238 (238)
  8 4WIZ_AR Coat protein; Virus, B  32.3      69 0.00089   19.7  11.4  185    1-192     6-227 (338)
  9 6IZL_A mud crab tombus-like vi  25.2   1E+02  0.0013   18.8  11.6  186    1-192     1-262 (337)
 10 2ZAH_A Coat protein; plant vir  24.9   1E+02  0.0013   18.8  11.4  183    1-192     5-216 (331)
 11 4V99_Jj Capsid protein; icosah  24.0 1.1E+02  0.0014   18.7  10.7  174    1-182     1-242 (242)
 12 6MRL_B p41; Plant virus, Tombu  22.5 1.2E+02  0.0015   18.5  10.7  183    1-192    37-275 (386)
 13 2TBV_B TOMATO BUSHY STUNT VIRU  20.1 1.4E+02  0.0018   18.1  10.9  183    1-192    42-282 (387)

No 1
>4NWW_A Capsid protein; beta barrel, virus; 3.75A {Orsay nodavirus}
Probab=81.73  E-value=4  Score=26.51  Aligned_cols=172  Identities=11%  Similarity=0.008  Sum_probs=91.8  Template_Neff=5.600

Q ss_pred             cccccccCCCCCCCCcCcceeEEEEEEccc----CCcceEeeHhheechHHHHHHHHHHHhccceEEehheEEEEECCcc
Q Q_5943908_1       3 GLSSKAQTMGREDDNRSSKMKVFHSELVYG----DNHNISIKKADLTGQHKMMLLLSSALRIGSVHMDVSRILVKWCPYI   78 (192)
Q Consensus         3 ~~~~~~~~~~~~~~~k~sK~~ay~~~~i~g----~~~n~~I~~~~fl~str~~L~l~sll~~~s~Ei~IkkI~i~WCP~V   78 (192)
                      |.++....       +=+.-.-...+...+    +.....+.+.-     ..+-+|.++. ..=-+.+++++.+.|-|.+
T Consensus         1 ~~~s~g~v-------~VsgrE~I~sVt~~as~s~G~~l~s~~InP-----~~fpwLs~iA-~~FqkYR~~sL~~~yvPsv   67 (358)
T 4NWW_A            1 GTTSSNSI-------LLKGCDRIVTVVDASTYDAGSAIVSIPITP-----DIAYRLGSTA-RTFQRIKYRSLKFRVNAQC   67 (358)
T ss_dssp             ---CCCCE-------EEEEEEEEEECSCCTTSCTTCEEEEEECCT-----TTSHHHHHHG-GGEEEEEEEEEEEEEEECC
T ss_pred             CccCCCeE-------EEEceEEEEEEEeCCCCCCCceEEEEEcCc-----cccHHHHHHh-hheeEEEEceEEEEEEeCC
Confidence            11111100       000000000011100    01111111111     1234676666 4557899999999999999


Q ss_pred             CCCCCceEEEEEEecCCCcccc----cc-ccceEEEEeeeecccceeEEEeCCeeEeecC-------CeEEEceEEEEec
Q Q_5943908_1      79 TPNMNTTIGITIKNNHHDDMSN----IN-DMSTYISVKGKMSEALQITWHPASTLVYKKG-------MSCIFPWVVDVDT  146 (192)
Q Consensus        79 EP~~~ssI~I~v~Y~~~~~~~~----~~-~D~tvv~~~G~iSE~L~Vvi~Pt~~li~~~~-------~a~~lPWsV~vet  146 (192)
                      --.....|.+-+.|+-.+..-.    .. -.+.--++.|.+++...+.+.+.+...|-..       .++.-|=.+.+-+
T Consensus        68 sTTtsGsVvmg~d~Dp~d~~ptg~ss~~~v~s~~~svs~pvW~~~sl~Ip~~~k~~Yt~~~~~sssD~r~~~~G~f~i~~  147 (358)
T 4NWW_A           68 ATTTAGGYVAGFVKDAADVLPTGTASIPYLMSNTGSFTQPWWKSTVHNVKIPQKLFYTEAPTRGADAVREYCPGQFHVLV  147 (358)
T ss_dssp             CTTCCCEEEEEEESCSSCCCCSGGGSHHHHHHSTTCEEEETTSCEEEEECCCSCCEESSCCSSSCCCGGGSCCCEEEEEE
T ss_pred             CCCCCeeEEEEEECCCCCCCCCCCCcHHHHHcCCCceEeCCccCeEEEeecCCccEEecCCCCCCCcccccCcEEEEEEE
Confidence            9999999999999987765332    11 1123345789999999888877666666542       2344555566665


Q ss_pred             CCeeccCCCcceeeEEEEEEEeeeecCCCCccccc----cccCCeeeecC
Q Q_5943908_1     147 GSTEQESGSPALGEIKIWCYFKMQYHKPSTRHIAR----AEIAPSIEWGN  192 (192)
Q Consensus       147 d~~~~~e~~~~lG~lkiWC~~~~~~~~~~~k~~s~----~y~~P~i~WsN  192 (192)
                      ++..   .+..+|  .+||.+++....+.......    .+.-|.-.|..
T Consensus       148 ~g~~---t~~~~G--~L~v~YdVeF~~P~~~~~~~~~~~~~i~~d~~~~~  192 (358)
T 4NWW_A          148 DSKP---SQICPV--TVDLEWVVELHDATFRKESDQTAISAIVADHTLNV  192 (358)
T ss_dssp             EECC---SSCCCE--EEEEEEEEEEEEECCCCCCBCCCBCCSCSSEEEEE
T ss_pred             ecCC---CCCccE--EEEEEEEEEEEecEecccccccccceeeecCCccc
Confidence            5432   244666  68888888887776554432    11112222221


No 2
>4NWV_A Capsid protein; beta barrel, VIRUS; 3.25A {Orsay virus}
Probab=60.80  E-value=18  Score=22.98  Aligned_cols=185  Identities=11%  Similarity=0.008  Sum_probs=95.8  Template_Neff=5.900

Q ss_pred             CCcccccccC-------CCCCCCCcCcceeEEEEEEcccCCcceEee----------HhheechHHHHHHHHHHHhccce
Q Q_5943908_1       1 MEGLSSKAQT-------MGREDDNRSSKMKVFHSELVYGDNHNISIK----------KADLTGQHKMMLLLSSALRIGSV   63 (192)
Q Consensus         1 ~~~~~~~~~~-------~~~~~~~k~sK~~ay~~~~i~g~~~n~~I~----------~~~fl~str~~L~l~sll~~~s~   63 (192)
                      |..-..++++       .|-.... ..+-..-+.+.|+|.+.-..|.          ...+.+.-..+-+|..+. ..=-
T Consensus        15 ~~~p~~~rr~~~~~r~~APVA~~~-~vr~~~~~sv~vsgrE~I~sVt~~~s~~aG~~l~s~~inPs~fprLs~iA-~~Yq   92 (391)
T 4NWV_A           15 VKQPSSHKRNNNPSRSVAPVAKAN-ALRTTSSNSILLKGCDRIVTVVDASTYDAGSAIVSIPITPDIAYRLGSTA-RTFQ   92 (391)
T ss_dssp             ---------------CCCCSCCCC-CCCCCCCCCEEEEEEEEEEECCCCTTCCTTCEEEEEECCGGGSHHHHHHH-TTCS
T ss_pred             CCCCccccCCCCCCCCCCceeece-eeeCCCCCeEEEEeeEEEEEEEcCCCCCCCceeEEEEcCcchhHhHHHHH-hhee
Confidence            1111111100       0000000 0000011222222222211111          012223333346666666 5556


Q ss_pred             EEehheEEEEECCccCCCCCceEEEEEEecCCCccccc--c---ccceEEEEeeeecccceeEEEeCCeeEeecCC----
Q Q_5943908_1      64 HMDVSRILVKWCPYITPNMNTTIGITIKNNHHDDMSNI--N---DMSTYISVKGKMSEALQITWHPASTLVYKKGM----  134 (192)
Q Consensus        64 Ei~IkkI~i~WCP~VEP~~~ssI~I~v~Y~~~~~~~~~--~---~D~tvv~~~G~iSE~L~Vvi~Pt~~li~~~~~----  134 (192)
                      ..+++++.+.|-|.+--.....|.+-+.|+-.+..-..  +   ....--++.|.+.+...+.+.+.++.++-..+    
T Consensus        93 kYR~~sL~~~yvPsvsTTTsG~Iima~d~Dp~d~~pt~~~s~~~l~s~~~svs~~vW~~~sL~Ip~~~kw~~t~~~a~s~  172 (391)
T 4NWV_A           93 RIKYRSLKFRVNAQCATTTAGGYVAGFVKDAADVLPTGTASIPYLMSNTGSFTQPWWKSTVHNVKIPQKLFYTEAPTRGA  172 (391)
T ss_dssp             EEEEEEEEEEEEECCCTTCCCEEEEEEESCSSCCCCCSTTHHHHHHHSTTCEEEETTSCEEEEEECTTCCEESSCCSSSC
T ss_pred             EEEEeEEEEEEEeCCCCCCCeeEEEEEECCCCCCCCCCCCcHHHHHcCCCcceeCcccCeEEEecCCCceEEeCCCCCCC
Confidence            88999999999999999999999999998887753210  1   11233456799999999999988888876554    


Q ss_pred             ---eEEEceEEEEecCCeeccCCCcceeeEEEEEEEeeeecCCCCcccccc----ccCCeeeecC
Q Q_5943908_1     135 ---SCIFPWVVDVDTGSTEQESGSPALGEIKIWCYFKMQYHKPSTRHIARA----EIAPSIEWGN  192 (192)
Q Consensus       135 ---a~~lPWsV~vetd~~~~~e~~~~lG~lkiWC~~~~~~~~~~~k~~s~~----y~~P~i~WsN  192 (192)
                         .+.-|=.+.+-+++..   ...++|  .+||.+++....+.......+    ...|.-.|.+
T Consensus       173 ~D~~~~~~G~f~v~~~g~~---s~~~~G--~L~v~Y~VeF~~P~~~~~~~~~~~~~i~~d~~~~~  232 (391)
T 4NWV_A          173 DAVREYCPGQFHVLVDSKP---SQICPV--TVDLEWVVELHDATFRKESDQTAISAIVADHTLNV  232 (391)
T ss_dssp             CCHHHHCSCEEEEEEEECC---SSCCCE--EEEEEEEEEEESCCCCCCCBCCCBCCSCSSEEEEE
T ss_pred             CccccccCEEEEEEEecCC---CCcccE--EEEEEEEEEEEcCEecCCccccccceeeecCeeee
Confidence               2222333333333321   223556  678888887777766655333    3334445554


No 3
>3ZXA_C CAPSID PROTEIN; VIRUS, SSRNA VIRUS, ICOSAHEDRAL VIRUS; 3.2A {TURNIP CRINKLE VIRUS}
Probab=56.78  E-value=22  Score=22.49  Aligned_cols=183  Identities=11%  Similarity=0.079  Sum_probs=87.7  Template_Neff=6.900

Q ss_pred             CCccccc---------------ccCCCCCCCCcCccee-EEEEEEcccCCcceEeeH--------hheechHHH---HHH
Q Q_5943908_1       1 MEGLSSK---------------AQTMGREDDNRSSKMK-VFHSELVYGDNHNISIKK--------ADLTGQHKM---MLL   53 (192)
Q Consensus         1 ~~~~~~~---------------~~~~~~~~~~k~sK~~-ay~~~~i~g~~~n~~I~~--------~~fl~str~---~L~   53 (192)
                      .-|+..-               ..........+| +|. +-.++.|++.++--.|..        ..|.+.-..   +-|
T Consensus        40 ~~G~g~y~~~~~~~~~~~~~apva~~~~~~~~~P-~~~~~~~~v~it~rE~i~~V~~~~~~~~~~~~~~inP~~~~tfPw  118 (347)
T 3ZXA_C           40 AMGIKLSPVAQPVQKVTRLSAPVALAYREVSTQP-RVSTARDGITRSGSELITTLKKNTDTEPKYTTAVLNPSEPGTFNQ  118 (347)
T ss_dssp             -------------CCCSCCCCCSCCCCCCCCCCC-CCCCSTTCCCCCCCCCCCCCCCCCSSSCCBCCBCCSSSCSTTCSS
T ss_pred             HhCCCCCCCCcccceeceeeCCCccceeecCCCC-eEEeCCCeEEEECeEEEEEeeeCCCCCCeeEEEEcCCCCcccChH
Confidence            0010000               000000111111 111 112333444443333321        122222221   335


Q ss_pred             HHHHHhccceEEehheEEEEECCccCCCCCceEEEEEEecCCCcccc--ccccceEEEEeeeecccceeEEEeCCee-Ee
Q Q_5943908_1      54 LSSALRIGSVHMDVSRILVKWCPYITPNMNTTIGITIKNNHHDDMSN--INDMSTYISVKGKMSEALQITWHPASTL-VY  130 (192)
Q Consensus        54 l~sll~~~s~Ei~IkkI~i~WCP~VEP~~~ssI~I~v~Y~~~~~~~~--~~~D~tvv~~~G~iSE~L~Vvi~Pt~~l-i~  130 (192)
                      |..+. ..=-+.++.++.+.|-|.+--....+|.+-..|+-.+..-.  ..-.+.--++.+..++.+.+.|-..... ++
T Consensus       119 Ls~iA-~~yekYr~~~l~f~y~p~~~ttt~G~V~ma~~~D~~d~~p~~k~~m~~~~~a~~~~~w~~~~l~i~~d~~~~y~  197 (347)
T 3ZXA_C          119 LIKEA-AQYEKYRFTSLRFRYSPMSPSTTGGKVALAFDRDAAKPPPNDLASLYNIEGCVSSVPWTGFILTVPTDSTDRFV  197 (347)
T ss_dssp             STTSS-SSBCCBCCSBCBCCBCCCSCSSSCCCCCCCCCSSCCSCCCSSSTTGGGSTTCCCCCTTSCBCCBCCCCCCCBCC
T ss_pred             HHHHH-HheeEEEEcEEEEEEEcCCCCCCCeeEEEEEeCCCCCCCCCCHHHHhcCCCcEEeCCcCCeEEEeecCCcccee
Confidence            55554 44457889999999999999888899999988887754322  1111344556777888777766433322 22


Q ss_pred             ecC---CeEEEceEEEEecCCeeccCCCcceeeEEEEEEEeeeecCCCCccccccccC-------Ce-eeecC
Q Q_5943908_1     131 KKG---MSCIFPWVVDVDTGSTEQESGSPALGEIKIWCYFKMQYHKPSTRHIARAEIA-------PS-IEWGN  192 (192)
Q Consensus       131 ~~~---~a~~lPWsV~vetd~~~~~e~~~~lG~lkiWC~~~~~~~~~~~k~~s~~y~~-------P~-i~WsN  192 (192)
                      ..+   .++.=+-.+.+-+++..    ...+|+  |||.+++....+..-.. .++..       |. +.++.
T Consensus       198 ~~~~~D~kl~d~G~f~iat~g~~----~~~~G~--L~v~Y~Vel~~P~~~~~-~q~~~~~~~~~Gp~~~~~~~  263 (347)
T 3ZXA_C          198 ADGISDPKLVDFGKLIMATYGQG----AAQLGE--VRVEYTVQLKNRTGSTS-AQIGDFAGVKDGPRLVSWSK  263 (347)
T ss_dssp             CSSCSCTTTSSSCCBCCBCCCCS----CCCCCC--CCCCCBCCCCSBCCCCC-CEECCCBTTBCCCCSCCSCC
T ss_pred             ecCCCCcccccceEEEEEEeCCC----CCceEE--EEEEEEEEEEecccCcc-ceeeccCCcccccceEEeec
Confidence            222   22233334444444332    267885  58888888877766544 34411       00 01110


No 4
>6MRM_C Capsid protein; RCNMV, virus; 2.9A {Red clover necrotic mosaic virus}
Probab=52.11  E-value=27  Score=21.95  Aligned_cols=183  Identities=9%  Similarity=0.029  Sum_probs=90.4  Template_Neff=7.000

Q ss_pred             CCc--ccccccCCC-----CCCCCcCcceeEEEEEEcccCCcceEeeH------------hheechHH---HHHHHHHHH
Q Q_5943908_1       1 MEG--LSSKAQTMG-----REDDNRSSKMKVFHSELVYGDNHNISIKK------------ADLTGQHK---MMLLLSSAL   58 (192)
Q Consensus         1 ~~~--~~~~~~~~~-----~~~~~k~sK~~ay~~~~i~g~~~n~~I~~------------~~fl~str---~~L~l~sll   58 (192)
                      +..  -+.+....|     .....+|   .+=..+.|++.++--.|..            ..|.+.-.   .+-||..+.
T Consensus        17 ~~~~~~~~~~~~APva~~~~~r~~~P---~~~~~~~i~~rE~l~~V~~s~~~~~~~g~~~~~~~lnP~~~~tfpwLs~iA   93 (339)
T 6MRM_C           17 NRTPNTSVKTVAIPFAKTQIIKTVNP---PPKPARGILHTQLVMSVVGSVQMRTNNGKSNQRFRLNPSNPALFPTLAYEA   93 (339)
T ss_dssp             --CCSCCCCCCCCCSCCCCCCCCCCC---CC-----CCCCEEEEEEECCSSCBSSSSSCTTCSBSCSSCTTTCTTHHHHH
T ss_pred             ccCCCCCCcceecccccceeeccCCC---CCCCcEEEEceEEEEEEEcCcccccCCCceeeEEEeCCCCcccchhHHHHH
Confidence            000  000000000     1112233   1112334444433323322            22222222   245777766


Q ss_pred             hccceEEehheEEEEECCccCCCCCceEEEEEEecCCCcccc--ccccceEEEEeeeecccceeEEEeCCeeEeec----
Q Q_5943908_1      59 RIGSVHMDVSRILVKWCPYITPNMNTTIGITIKNNHHDDMSN--INDMSTYISVKGKMSEALQITWHPASTLVYKK----  132 (192)
Q Consensus        59 ~~~s~Ei~IkkI~i~WCP~VEP~~~ssI~I~v~Y~~~~~~~~--~~~D~tvv~~~G~iSE~L~Vvi~Pt~~li~~~----  132 (192)
                       ..=-+.++.++.+.|-|.+--.....|.+-..|+-.+..-.  .+--..--++.+.+++.+.+.|-.....-|-+    
T Consensus        94 -~~f~kYr~~~l~~~y~p~~~tT~~G~V~ma~d~D~~d~~p~s~~~l~~~~~~~s~~~w~~~~l~i~~d~~~ry~~~~~~  172 (339)
T 6MRM_C           94 -ANYDMYRLKKLTLRYVPLVTVQNSGRVAMIWDPDSQDSAPQSRQEISAYSRSVSTAVYEKCSLTIPADNQWRFVADNTT  172 (339)
T ss_dssp             -TTEEEECCCCEEECCEECSCTTCCCEEEEEEESCSSCCCCSSSHHHHCSSSEECCBSSSCCCEEECCCCCCEECCCSCC
T ss_pred             -hheeEEEEcEEEEEEEeCCCCCCCceEEEEEcCCCCCCCCCCHHHHHhcCCcEEeCccccEEEEeeCCccceeEecCCC
Confidence             45678999999999999999999999999999997763211  11113566788889998877764433332221    


Q ss_pred             -CCeEEEceEEEEecCCeeccCCCcceeeEEEEEEEeeeecCCCCccccccccC---C---eeeecC
Q Q_5943908_1     133 -GMSCIFPWVVDVDTGSTEQESGSPALGEIKIWCYFKMQYHKPSTRHIARAEIA---P---SIEWGN  192 (192)
Q Consensus       133 -~~a~~lPWsV~vetd~~~~~e~~~~lG~lkiWC~~~~~~~~~~~k~~s~~y~~---P---~i~WsN  192 (192)
                       |.++.=+-.+.+-+++..   ....+|+  +||.+++..+.+.......+...   +   ...-+.
T Consensus       173 ~D~rl~d~G~f~iat~g~~---~~~~~G~--l~v~Y~V~f~~P~~~~~~~~~~~~~~~~~~~~~~G~  234 (339)
T 6MRM_C          173 VDRKLVDFGQLLFVTHSGS---DGIETGD--IFLDCEVEFKGPQPTASIVQKTVIDLGGTLTSFEGP  234 (339)
T ss_dssp             SCTTSSSCCEECCEEESCC---CSSEEEE--EEEECCCCEEEECSCCCCCCEEEECTTCCCEEESSC
T ss_pred             CCccceecEEEEEEEEcCC---CCcceEE--EEEEEEEEEECCccCcccceeeEeecCCCcccccCC
Confidence             222222344455554442   3456777  58888887776655443222221   0   000000


No 5
>4Y5Z_Q Immunoglobulin G-binding protein A,Coat protein; coat protein, chimeric VLP, in vitro assembly, VIRUS; HET: SO4; 2.95A {Staphylococcus aureus} SCOP: b.121.4.7
Probab=51.57  E-value=28  Score=21.89  Aligned_cols=177  Identities=10%  Similarity=0.030  Sum_probs=45.9  Template_Neff=7.500

Q ss_pred             CCcccccccC-CCCCCCCcCccee-EEEEEEcccCCcceEe------eHhheechHHHH-HHHHHHHhccceEEehheEE
Q Q_5943908_1       1 MEGLSSKAQT-MGREDDNRSSKMK-VFHSELVYGDNHNISI------KKADLTGQHKMM-LLLSSALRIGSVHMDVSRIL   71 (192)
Q Consensus         1 ~~~~~~~~~~-~~~~~~~k~sK~~-ay~~~~i~g~~~n~~I------~~~~fl~str~~-L~l~sll~~~s~Ei~IkkI~   71 (192)
                      -|+..-.+.+ ...+...+|..-. +-..+.|+|...-..|      ....|.+.-..+ -+|..+. ..=-..++.++.
T Consensus        60 ~~~~k~n~~~~p~~~~~~~P~~~~~~~~~~~i~~~E~i~~v~~~~~~~~~~~~~~p~~f~p~Ls~~A-~~y~kYr~~~l~  138 (282)
T 4Y5Z_Q           60 SEAKKLNESQAPKEGSELEMAVSSSRGGITVLTHSELSAEIGVTDSIVVSSELVMPYTVGTWLRGVA-ANWSKYSWLSVR  138 (282)
T ss_dssp             --------------------------CCCEEEEEEEEEEEEEECSSCEEEEEECSHHHHCHHHHHHH-TTEEEEEEEEEE
T ss_pred             HHHHhhccCCCCCCcccccccEeecCCCEEEEEceEEEEEeecCCCceeeeeecChhhHHHHHHHHH-HheeEEEEeEEE
Confidence            0000000000 0000001111000 0000111110000000      011133333344 3444333 233344555555


Q ss_pred             EEECCccCCCCCceEEEEEEecCCCcccc--ccccceEEEEeeeecccce----eE--------------EEe---CCee
Q Q_5943908_1      72 VKWCPYITPNMNTTIGITIKNNHHDDMSN--INDMSTYISVKGKMSEALQ----IT--------------WHP---ASTL  128 (192)
Q Consensus        72 i~WCP~VEP~~~ssI~I~v~Y~~~~~~~~--~~~D~tvv~~~G~iSE~L~----Vv--------------i~P---t~~l  128 (192)
                      +.|-|.+--.....|.+-+.|+-.+..-.  ..-.+.--++.|.+.+.+.    +.              +-|   ..+.
T Consensus       139 ~~y~p~~~ttt~G~v~~~~~~D~~d~~p~~~~~l~~~~~~~~~~vw~~~~~~~~l~ip~~~~~~~~~~~~~d~~~~~~~w  218 (282)
T 4Y5Z_Q          139 YTYIPSCPSSTAGSIHMGFQYDMADTVPVSVNQLSNLRGYVSGQVWSGSAGLCFINGTRCSDTSTAISTTLDVSKLGKKW  218 (282)
T ss_dssp             EEEEECSCTTCCCEEEEEEECCTTSCCCCSHHHHTTSEEEEEEETTCCGGGHHHHTTCCCCCCTTCCEEECCGGGSSCSC
T ss_pred             EEEEcCCCCCCCeEEEEEEeCCCCCCCCCCHHHHHccCCcEEecCccCcCceeeeecccCCCCCceeEEEEehhhcCcce
Confidence            55555555555555555555544433210  0011233334444444331    10              111   1112


Q ss_pred             Eee--------------cCCeEEEceEEEEecCCeeccCCCcceeeEEEEEEEeeeecCCCCccccc
Q Q_5943908_1     129 VYK--------------KGMSCIFPWVVDVDTGSTEQESGSPALGEIKIWCYFKMQYHKPSTRHIAR  181 (192)
Q Consensus       129 i~~--------------~~~a~~lPWsV~vetd~~~~~e~~~~lG~lkiWC~~~~~~~~~~~k~~s~  181 (192)
                      ++.              .+..+.-|=.+.+-+++.. ......+|+|  ||.+++....+....-.-
T Consensus       219 y~~~~~~~~~~~~~~~~~d~~~~~~G~~~i~~~g~~-~~~~~~~G~l--~v~Y~Vel~~P~~~~~~~  282 (282)
T 4Y5Z_Q          219 YPYKTSADYATAVGVDVNIATPLVPARLVIALLDGS-SSTAVAAGRI--YCTYTIQMIEPTASALNN  282 (282)
T ss_dssp             EECCCHHHHHHHHHHCGGGGTTTCCCEEEEEEECCS-CSSCEEEEEE--EEEEEEEEEEECC-----
T ss_pred             eeecCCcccccccCCCcCCCCccccEEEEEEEecCC-CCCcceeEEE--EEEEEEEEEcCcchhcCC
Confidence            221              0111222333333333321 1122333332  333333332222111111


No 6
>1OPO_B Coat protein; PLANT VIRUS, CARMOVIRUS, VIRUS/VIRAL PROTEIN, TOMATO BUSHY STUNT VIRUS, Icosahedral virus, Virus; HET: SO4; 3.2A {Carnation mottle virus} SCOP: b.121.4.7
Probab=50.02  E-value=30  Score=21.72  Aligned_cols=187  Identities=11%  Similarity=0.092  Sum_probs=92.3  Template_Neff=7.000

Q ss_pred             CCc---ccccccCC------------------CCCCCCcCcce-----eEEEEEEcccCCcceEeeH--------hheec
Q Q_5943908_1       1 MEG---LSSKAQTM------------------GREDDNRSSKM-----KVFHSELVYGDNHNISIKK--------ADLTG   46 (192)
Q Consensus         1 ~~~---~~~~~~~~------------------~~~~~~k~sK~-----~ay~~~~i~g~~~n~~I~~--------~~fl~   46 (192)
                      -.|   |+.+.+..                  .+..++++..-     .+-..+.|++.+.--.|..        ..|.+
T Consensus        30 ~~gw~~l~~~qk~~~~~~~~~~~~~~v~~~~~~r~~~~~~r~~~~~p~~~~~~v~it~~E~l~~V~~~~~~~~~~~~~~i  109 (348)
T 1OPO_B           30 TRGWASLSTNQKRRAEMLAGYTPAILAFTPRRPRMTNPPPRTSRNSPGQAGKSMTMSKTELLSTVKGTTGVIPSFEDWVV  109 (348)
T ss_dssp             ----------------------------------------------------CEEEEEEEEEEEEECCCSSSCCEEECCC
T ss_pred             hhhHhhcCHHHHHHHHhhhCCCCcccccccCCCCCCCCCCccCCCCCCCCCCeEEEECeEEEEEEEcCCCCCcceeEEEe
Confidence            001   11110000                  00011111000     1122333333322222211        12222


Q ss_pred             hHH---HHHHHHHHHhccceEEehheEEEEECCccCCCCCceEEEEEEecCCCcccc--ccccceEEEEeeeecccceeE
Q Q_5943908_1      47 QHK---MMLLLSSALRIGSVHMDVSRILVKWCPYITPNMNTTIGITIKNNHHDDMSN--INDMSTYISVKGKMSEALQIT  121 (192)
Q Consensus        47 str---~~L~l~sll~~~s~Ei~IkkI~i~WCP~VEP~~~ssI~I~v~Y~~~~~~~~--~~~D~tvv~~~G~iSE~L~Vv  121 (192)
                      .-.   .+-+|.++. ..=-..++.++.+.|-|.+--.....|.+-..|+..+..-.  .+--+..-.+.+.+.+.+.+.
T Consensus       110 nP~~~~~fp~Ls~iA-~~f~kYr~~~l~~~yvp~~~Ttt~G~V~~~~d~D~~D~~p~s~~~l~~~~~~~~~~~w~~~~l~  188 (348)
T 1OPO_B          110 SPRNVAVFPQLSLLA-TNFNKYRITALTVKYSPACSFETNGRVALGFNDDASDTPPTTKVGFYDLGKHVETAAQTAKDLV  188 (348)
T ss_dssp             CTTCTTTCHHHHHHH-TTEEEEEEEEEEEEEEECSCTTCCCEEEEEEESCTTSCCCSSTTTTTTSSEEEEEETTSCEEEE
T ss_pred             cCCchHhcHHHHHHH-HhceEEEEeEEEEEEEeCCCCCCCceEEEEEEcCCCCCCCCCHHHHHhcCCcEEeCCcCCeeEE
Confidence            222   245777776 45568999999999999999999999999999998775432  222356788899999998888


Q ss_pred             EEeCCeeEeecCC-----eEEEceEEEEecCCeeccCCCcceeeEEEEEEEeeeecCCCCccccccccCCe---------
Q Q_5943908_1     122 WHPASTLVYKKGM-----SCIFPWVVDVDTGSTEQESGSPALGEIKIWCYFKMQYHKPSTRHIARAEIAPS---------  187 (192)
Q Consensus       122 i~Pt~~li~~~~~-----a~~lPWsV~vetd~~~~~e~~~~lG~lkiWC~~~~~~~~~~~k~~s~~y~~P~---------  187 (192)
                      |.+....-|-.+.     ++.=|-.+.+-+++...  ....+|+|  ||.+++....+.......+.....         
T Consensus       189 i~~d~~~ry~~~~~~~D~kl~d~G~~~v~~~g~~~--~~~~~G~l--~i~Y~Vef~~P~~~~~~~~~~~~~~~~gp~~~~  264 (348)
T 1OPO_B          189 IPVDGKTRFIRDSASDDAKLVDFGRIVLSTYGFDK--ADTVVGEL--FIQYTIVLSDPTKTAKISQASNDKVSDGPTYVV  264 (348)
T ss_dssp             ECCCCCCEECCCSTTSCHHHHCCCEEEEEEECCSS--SSCEEEEE--EEEEEEEEEEECSCCCCCEETTCGGGCSCCSEE
T ss_pred             eecCCCceeEeCCCCCccccccceEEEEEEecCCC--CCceeEEE--EEEEEEEEeCCeeccccccccCCCCcccCceee
Confidence            8665544444332     11234455555554422  46788866  666666555554444333332210         


Q ss_pred             eeecC
Q Q_5943908_1     188 IEWGN  192 (192)
Q Consensus       188 i~WsN  192 (192)
                      +..+.
T Consensus       265 ~~~~~  269 (348)
T 1OPO_B          265 PSVNG  269 (348)
T ss_dssp             EEEET
T ss_pred             eeecC
Confidence            01110


No 7
>1F2N_C CAPSID PROTEIN; PLANT VIRUS, CAPSID PROTEIN, COAT PROTEIN, BETA-ANNULUS, DOMAIN SWAPPING, Icosahedral virus, Virus; 2.8A {Rice yellow mottle virus} SCOP: b.121.4.7
Probab=33.53  E-value=65  Score=19.87  Aligned_cols=172  Identities=12%  Similarity=0.082  Sum_probs=38.1  Template_Neff=8.000

Q ss_pred             CCcc-----------cccccCCCCC-----CCCc-----CcceeEEEEEEcc----cCC-cceEeeHhheechHHHHHHH
Q Q_5943908_1       1 MEGL-----------SSKAQTMGRE-----DDNR-----SSKMKVFHSELVY----GDN-HNISIKKADLTGQHKMMLLL   54 (192)
Q Consensus         1 ~~~~-----------~~~~~~~~~~-----~~~k-----~sK~~ay~~~~i~----g~~-~n~~I~~~~fl~str~~L~l   54 (192)
                      |...           +.+....|-.     ....     ......-+.+.|.    +.. ..    ...|.+.-..+-+|
T Consensus        13 ~~~~~~~~~~~~r~~~~~~v~aP~a~~~~~~~~~p~~~~~~~~~v~~~E~l~~v~~~~~~~~----~~~~~l~P~~fp~L   88 (238)
T 1F2N_C           13 QQGKRKSRRPRGRSAEPQLQRAPVAQASRISGTVPGPLSSNTWPLHSVEFLADFKRSSTSAD----ATTYDCVPFNLPRV   88 (238)
T ss_dssp             --------------CCCCCCCCCSCCCCEEESSCSSTTCSSEEEEEEEEEEEEEEECSSCCC----CEEEECSGGGSHHH
T ss_pred             cCCCCCCCCCCCCCCCCceeeCCceeeeEecCCCCccccCCeeEEEeeEEEEEeecCCCCcc----ceeEEeCCccchhH
Confidence            0000           0000000000     0000     0001111111000    000 00    00111111222333


Q ss_pred             HHHHhccceEEehheEEEEECCccCCCCCceEEEEEEecCCCcccc-cccc-ceEEEEeeeec---------------cc
Q Q_5943908_1      55 SSALRIGSVHMDVSRILVKWCPYITPNMNTTIGITIKNNHHDDMSN-INDM-STYISVKGKMS---------------EA  117 (192)
Q Consensus        55 ~sll~~~s~Ei~IkkI~i~WCP~VEP~~~ssI~I~v~Y~~~~~~~~-~~~D-~tvv~~~G~iS---------------E~  117 (192)
                      ..+. ..=-...+.++.+.|-|.+--.....|.+-+.|+-.+..-. ..+- ...-++.|.+.               +.
T Consensus        89 ~~iA-~~y~kyr~~~l~~~y~p~~~ttt~G~v~m~~~~D~~d~~p~~~~~~~~~~~~~~~~vw~~~~g~~~~~~~~~~~~  167 (238)
T 1F2N_C           89 WSLA-RCYSMWKPTRWDVVYLPEVSATVAGSIEMCFLYDYADTIPRYTGKMSRTAGFVTSSVWYGAEGCHLLSGGSARNA  167 (238)
T ss_dssp             HHHH-TTEEEEEESCCEEEEEECSCTTCCCEEEEEEESCTTSCCCCSHHHHHTSTTCEEEETTCCGGGGGTSSSCCGGGC
T ss_pred             HHHH-HhceEEEecEEEEEEeeCCCCCCCeEEEEEEeCCCCCCCCCcHHHHHccCCcEEeccccCcccceecCCCCCCcc
Confidence            3333 22233444444444444444444444444444443322100 0000 00001111111               11


Q ss_pred             ceeEEEeCCeeEeec-----------CCeEEEceEEEEecCCeeccCCCcceeeEEEEEEEeeeecCCCCccccc
Q Q_5943908_1     118 LQITWHPASTLVYKK-----------GMSCIFPWVVDVDTGSTEQESGSPALGEIKIWCYFKMQYHKPSTRHIAR  181 (192)
Q Consensus       118 L~Vvi~Pt~~li~~~-----------~~a~~lPWsV~vetd~~~~~e~~~~lG~lkiWC~~~~~~~~~~~k~~s~  181 (192)
                      +.+.+.+.+.-.|..           +..+.-|=.+.+-+++.  ...+..+|+  |||.+++....+..-.-.-
T Consensus       168 ~~~~l~~~~~~~~~~~~~~~~~~~~~d~~~~~~G~~~i~~~~~--~~~~~~~G~--l~v~Y~Vel~~P~~~~~n~  238 (238)
T 1F2N_C          168 VVASMDCSRVGWKRVTSSIPSSVDPNVVNTILPARLAVRSSIK--PTVSDTPGK--LYVIASMVLRDPVDPTLNT  238 (238)
T ss_dssp             EEEECCCTTCCCEECCSCCCCSSCHHHHHTTCSCEEEEEESSC--CSSCEEEEE--EEEEEEEEEEEECCTTTCC
T ss_pred             EEEEEeCCCCccEEeccCCCCCCCcchhcccccEEEEEEecCC--CCCCCCCeE--EEEEEEEEEECCCCcccCC
Confidence            112222211100000           00112222233333222  011122222  3333333333322211111


No 8
>4WIZ_AR Coat protein; Virus, Betanodavirus; HET: CA; 3.6A {Epinephelus coioides nervous necrosis virus}
Probab=32.31  E-value=69  Score=19.73  Aligned_cols=185  Identities=14%  Similarity=0.081  Sum_probs=87.3  Template_Neff=7.200

Q ss_pred             CCccc------ccccCCCC------------CCCCcCccee----EEEEEEcccCCcc----eE-e------eHhheech
Q Q_5943908_1       1 MEGLS------SKAQTMGR------------EDDNRSSKMK----VFHSELVYGDNHN----IS-I------KKADLTGQ   47 (192)
Q Consensus         1 ~~~~~------~~~~~~~~------------~~~~k~sK~~----ay~~~~i~g~~~n----~~-I------~~~~fl~s   47 (192)
                      |..+.      .+.+|...            .+-+..+...    +-..+.+.|...-    +. .      ...++.+.
T Consensus         6 ~~~~~~~~~~k~~~~q~~~r~~~Rrr~~~~~APva~~~~~~~~~~~~~~~~~sG~d~l~~~~v~~~~~~~g~~l~~~~in   85 (338)
T 4WIZ_AR           6 EKKLAKPATTKAANPQPRRRANNRRRSNRTDAPVSKASTVTGFGRGTNDVHLSGMSRISQAVLPAGTGTDGYVVVDATIV   85 (338)
T ss_pred             ccccccCccCCCCCCCCCCCCCCCCCCCCCCCCcceeeeeccCCCCCceEEEecceEEEEEECCCCCCCCCceEEEEEcC
Confidence            00000      00000000            0000000000    0011111111100    00 0      01112222


Q ss_pred             HHHHHHHHHHHhccceEEehheEEEEECCccCCCCCceEEEEEEecCCCccccccc-cceEEEEeeeecccceeEEEeCC
Q Q_5943908_1      48 HKMMLLLSSALRIGSVHMDVSRILVKWCPYITPNMNTTIGITIKNNHHDDMSNIND-MSTYISVKGKMSEALQITWHPAS  126 (192)
Q Consensus        48 tr~~L~l~sll~~~s~Ei~IkkI~i~WCP~VEP~~~ssI~I~v~Y~~~~~~~~~~~-D~tvv~~~G~iSE~L~Vvi~Pt~  126 (192)
                      -..+-+|..+. ..=...++.++.+.|-|.+--.....|.+-+.|+-.+....... ...--++.+.+.|...+.+-...
T Consensus        86 P~~~prL~~~A-~~y~ryr~~~l~~~~~p~~~Ttt~G~vv~~~~~Dp~d~~~s~~~l~a~~~s~~~~~w~~~~l~i~~~~  164 (338)
T 4WIZ_AR          86 PDLLPRLGHAA-RIFQRYAVETLEFEIQPMCPANTGGGYVAGFLPDPTDNDHTFDALQATRGAVVAKWWESRTVRPQYTR  164 (338)
T ss_pred             cccCHHHHHHH-HhheEEEEeEEEEEEEcCCCcCCCeeEEEEEeCCCCCCCCcHHHHHcCCCcEEEccccCeeecCcCCC
Confidence            23334555444 33457889999999999999999999999998887765422221 12344567777787766654322


Q ss_pred             eeEee---cCCeEEEceEEEEecCCeeccCCCcceeeEEEEEEEeeeecCCCCccccccccCCeeeecC
Q Q_5943908_1     127 TLVYK---KGMSCIFPWVVDVDTGSTEQESGSPALGEIKIWCYFKMQYHKPSTRHIARAEIAPSIEWGN  192 (192)
Q Consensus       127 ~li~~---~~~a~~lPWsV~vetd~~~~~e~~~~lG~lkiWC~~~~~~~~~~~k~~s~~y~~P~i~WsN  192 (192)
                      ...|-   .+..+.-|=.+-+-+++...   +.  +.+.|||..++....+.-... .....|...|.+
T Consensus       165 ~~~~~~~g~d~~~~spG~~~v~~~g~~~---~~--~~~~v~v~Y~Vef~~P~~~~~-~~~~~~~~~~~~  227 (338)
T 4WIZ_AR         165 TLLWTSSGKEQRLTSPGRLILLCVGNNT---DV--VNVSVLCRWSVRLSVPSLENP-EETTAPIMTQGS  227 (338)
T ss_pred             ceeeecCCCcccccccEEEEEEEecCCC---CC--cEEEEEEEEEEEEEcccCCCC-Cccccccccccc
Confidence            22221   11122233333333332211   11  234899999999888776554 345668888888


No 9
>6IZL_A mud crab tombus-like virus; virus, capsid; 3.3A {Wenzhou tombus-like virus 18}
Probab=25.20  E-value=1e+02  Score=18.84  Aligned_cols=186  Identities=13%  Similarity=0.108  Sum_probs=85.4  Template_Neff=6.600

Q ss_pred             CCccccc---------ccC-----------------------CCCCCCCcCcceeEEEEEEcccCCc-------------
Q Q_5943908_1       1 MEGLSSK---------AQT-----------------------MGREDDNRSSKMKVFHSELVYGDNH-------------   35 (192)
Q Consensus         1 ~~~~~~~---------~~~-----------------------~~~~~~~k~sK~~ay~~~~i~g~~~-------------   35 (192)
                      |.+-.++         +.+                       .+-.....-.+-.+-.++.|+|.+.             
T Consensus         1 ~~~~~~~~~~~~~~~~~~~~~~~~~rr~~~~~~~~~~~~~~~~p~~~~~~~~R~~~~~s~~vsg~E~l~~V~~~a~~~~~   80 (337)
T 6IZL_A            1 MTGSNRRANAGRKTQPKPQRKPRAPRRPKVQNAPRIQQGGGPVPLLESNSNMRQMHNGMTRVVGSDYLGVVSVAGNPADA   80 (337)
T ss_dssp             --------------------------------------------------CCCCCCCCCEEEEEEEEEEEEEECSSCCSS
T ss_pred             CCccccccCCCCCCCCCCCCCCCCCCCcccCCCCCccccCcccccCCCCceecccCCCeEEEEeeEEEEEEEeCCCCccc
Confidence            1100000         000                       0000000000000111112222211             


Q ss_pred             ------ceEeeHhheechHHHHHHHHHHHhccceEEehheEEEEECCccCCCCCceEEEEEEecCCCcc---cc-cc---
Q Q_5943908_1      36 ------NISIKKADLTGQHKMMLLLSSALRIGSVHMDVSRILVKWCPYITPNMNTTIGITIKNNHHDDM---SN-IN---  102 (192)
Q Consensus        36 ------n~~I~~~~fl~str~~L~l~sll~~~s~Ei~IkkI~i~WCP~VEP~~~ssI~I~v~Y~~~~~~---~~-~~---  102 (192)
                            .+.|+-.++.+     -+|..+. ..=-...+.++.+.|-|.+--.....|.+-+.|+-.+..   .+ .+   
T Consensus        81 ~g~~~~~~~inPs~~~~-----pwLs~iA-~~yekYr~~~l~~~yvP~~sTTt~G~V~m~fd~Dp~d~~~~p~~~~~~~~  154 (337)
T 6IZL_A           81 AAKVRKVLSVSPSSFPG-----TRLTQMS-DLWERYVFRQFRVRYVPSVPNTLACQVMVYQDTDPQDDPTAIKDADALLR  154 (337)
T ss_dssp             SSSEEEEEEESSSCSSS-----CHHHHHH-TTBSEEEEEEEEEEEECCSCTTCBCEEEEEEESCTTCCGGGCSCHHHHHH
T ss_pred             ccceEEEEEcCcccCCC-----chHHHHh-hheeEEEEeEEEEEEEeCCCCCCCeeEEEEEeCCCCCCCccCCCHHHHHH
Confidence                  22333333332     1455554 333578889999999999999999999999988887742   11 11   


Q ss_pred             -ccceEEEEeeeecccceeEEEe--CCeeEeecCC----eEEEceEEEEecCCeeccCC-Ccc---eeeEEEEEEEeeee
Q Q_5943908_1     103 -DMSTYISVKGKMSEALQITWHP--ASTLVYKKGM----SCIFPWVVDVDTGSTEQESG-SPA---LGEIKIWCYFKMQY  171 (192)
Q Consensus       103 -~D~tvv~~~G~iSE~L~Vvi~P--t~~li~~~~~----a~~lPWsV~vetd~~~~~e~-~~~---lG~lkiWC~~~~~~  171 (192)
                       ....--+..+.+.+...+.|-+  ....++..+.    ++.-|-.+.+-+++...... ..+   +....|||.+++..
T Consensus       155 ~l~a~~~s~~~~vw~~~~l~Ip~~~~~r~y~td~~~~D~r~~d~G~~~v~~~g~~~~~~g~~vt~~~~~G~l~v~YtV~f  234 (337)
T 6IZL_A          155 QATAQTGSQQWNFNSAKVIHLAKRSDNQLYYTGPVKENPRFNQQGVVYFIQVSQALDMNGKPLTADMECGSLYVDWVIDF  234 (337)
T ss_dssp             HHHHSTTCEEEETTSCEEECCCCCTTCCCEECSSCSSCGGGTCSEEEEEEECSCCBCGGGCBCSSCEEEEEEEEEEEEEE
T ss_pred             HHHhCCCceEEcCCCCcEEEeecCCCCCeEEcCCCCCCcccccCeEEEEEEecCCcCCCCCcCcccceeeEEEEEEEEEE
Confidence             0111224567888888777732  3455554332    34577888888887654321 213   23445677777766


Q ss_pred             cCCCCccccccc-------cCCeeeecC
Q Q_5943908_1     172 HKPSTRHIARAE-------IAPSIEWGN  192 (192)
Q Consensus       172 ~~~~~k~~s~~y-------~~P~i~WsN  192 (192)
                      ..+..-....+.       ....+.+..
T Consensus       235 ~~P~~~~~~~~~~~~gp~y~~~~~~~~~  262 (337)
T 6IZL_A          235 QTPQVNPSAVEARLPSAGFFTRQILVND  262 (337)
T ss_dssp             CSBCCCTTC-------------------
T ss_pred             ECCccCccccccccCCCceEEEEEEcee
Confidence            665554444433       333333211


No 10
>2ZAH_A Coat protein; plant virus, coat protein, b-annulus, tombusvirus, carmovirus, fungal vector, MNSV, Capsid protein, ICOSAHEDRAL VIRUS, VIRUS; 2.81A {Melon necrotic spot virus}
Probab=24.86  E-value=1e+02  Score=18.79  Aligned_cols=183  Identities=11%  Similarity=0.028  Sum_probs=89.6  Template_Neff=6.900

Q ss_pred             CCcccccccCCC-----CCCCCcCccee-EEEEEEcccCCcceEe-------------eHhheechHH---HHHHHHHHH
Q Q_5943908_1       1 MEGLSSKAQTMG-----REDDNRSSKMK-VFHSELVYGDNHNISI-------------KKADLTGQHK---MMLLLSSAL   58 (192)
Q Consensus         1 ~~~~~~~~~~~~-----~~~~~k~sK~~-ay~~~~i~g~~~n~~I-------------~~~~fl~str---~~L~l~sll   58 (192)
                      -.|-+.+....|     .....+| +|. +-..+.|++.++=..|             ....|.+.-.   .+-||..+.
T Consensus         5 ~~~~~~~~~~APva~~~~i~~~~P-~~~~~~~~~~I~~rE~l~~V~~~~~~~v~~G~~~~~~~~lnP~~~~~fpwLs~iA   83 (331)
T 2ZAH_A            5 TEGAKPGAISAPVAISRRVAGMKP-RFVRSEGSVKIVHREFIASVLPSNDLTVNNGDVNIGKYRVNPSNNALFTWLQGQA   83 (331)
T ss_dssp             --------------------------------CEEEEEEEEEEEECCCSSCEEGGGCCCTTTEESCTTCTTTCTTTHHHH
T ss_pred             cCCCCCCccccceeeeeeecCCCC-EEEecCCEEEEEceEEEEEeecCCCcccCCCCceeeEEEcCCcccccChhHHHHH
Confidence            111111000000     0001111 011 1112222222211111             1122333221   245777776


Q ss_pred             hccceEEehheEEEEECCccCCCCCceEEEEEEecCCCccc-c-ccccceEEEEeeeecccceeEEEeCCeeEeecC---
Q Q_5943908_1      59 RIGSVHMDVSRILVKWCPYITPNMNTTIGITIKNNHHDDMS-N-INDMSTYISVKGKMSEALQITWHPASTLVYKKG---  133 (192)
Q Consensus        59 ~~~s~Ei~IkkI~i~WCP~VEP~~~ssI~I~v~Y~~~~~~~-~-~~~D~tvv~~~G~iSE~L~Vvi~Pt~~li~~~~---  133 (192)
                       ..=-+.++.++.+.|-|.+--.....|.+-..|+-.+..- + .+--..--++.+..++...+.|.+.....|-.+   
T Consensus        84 -~~y~kYr~~~l~~~y~p~~~ttt~G~V~m~~d~D~~d~~p~s~~~l~~~~~~~s~~~w~~~~l~i~~d~~~~y~~~~~~  162 (331)
T 2ZAH_A           84 -QLYDMYRFTRLRFTYIPTTGSTSTGRVSILWDRDSQDPLPIDRAAISSYAHYADSAPWAENVLVVPCDNTWRYMNDTNA  162 (331)
T ss_dssp             -TTBSEEEEEEEEEEEEECSCTTCCCEEEEEEESCTTSCCCCSHHHHTCCSEEEEECTTSCEEEEECCCSCCEECCCSCC
T ss_pred             -HhceEEEEeEEEEEEEeCCCCCCCcEEEEEEeCCCCCCCCCCHHHHHhcCCcEEeCCccCeEEEccCCCcceeeeCCCC
Confidence             4556899999999999999999999999999998877421 1 122246678889999998888865554444432   


Q ss_pred             --CeEEEceEEEEecCCeeccCCCcceeeEEEEEEEeeeecCCCCccccccccCCeeeecC
Q Q_5943908_1     134 --MSCIFPWVVDVDTGSTEQESGSPALGEIKIWCYFKMQYHKPSTRHIARAEIAPSIEWGN  192 (192)
Q Consensus       134 --~a~~lPWsV~vetd~~~~~e~~~~lG~lkiWC~~~~~~~~~~~k~~s~~y~~P~i~WsN  192 (192)
                        .++.=|=.+.+-+++..   ....+|+|  |+.+++....+..-...-++-  +-.++.
T Consensus       163 ~D~kl~d~G~~~iat~g~~---~~~~~G~l--~i~Y~V~l~~P~~~~~~~~~~--~~~~~~  216 (331)
T 2ZAH_A          163 VDRKLVDFGQFLFATYSGA---GATAHGDL--YVEYAVEFKDPQPIAGMVCMF--DRLVSF  216 (331)
T ss_dssp             SSGGGTSCCEEEEEEESCC---SSSCCEEE--EEEEEEEECCBCSCCCSEEEE--EECTTT
T ss_pred             CCcccccceEEEEEEecCC---CCceeEEE--EEEEEEEEECCccCcccceee--eeeeee
Confidence              12222334445444442   35677875  777777776665544333310  011111


No 11
>4V99_Jj Capsid protein; icosahedral virus, tombusviridae, RNA hairpin, virus coat protein, Swiss jelly roll fold, VIRUS-RNA complex; HET: CA; 2.9A {Panicum mosaic virus}
Probab=23.97  E-value=1.1e+02  Score=18.67  Aligned_cols=174  Identities=7%  Similarity=-0.001  Sum_probs=46.7  Template_Neff=7.800

Q ss_pred             CCcc--------cccccC---------CCCCC------------CCcCcceeEEEEEEcccCCcceEee-------Hhhe
Q Q_5943908_1       1 MEGL--------SSKAQT---------MGRED------------DNRSSKMKVFHSELVYGDNHNISIK-------KADL   44 (192)
Q Consensus         1 ~~~~--------~~~~~~---------~~~~~------------~~k~sK~~ay~~~~i~g~~~n~~I~-------~~~f   44 (192)
                      |.+-        ..+.+|         .+.+.            ...|.--..=..+.|+|.+.-..|.       ...|
T Consensus         1 ~~~~~~~~~~~~~~~~~~~~~~~~~~~rs~~~~saPva~~~~v~~~~P~~~~~~~~~~i~~~E~l~~v~~~~~~~~~~~~   80 (242)
T 4V99_Jj           1 MNRNGATPTRGRGKRAIPNPPRRRARGKSVERGSTPLQYVTTLGPSRPRMGQGQGWQKLSHEEIILQVNSSTAADTIQTI   80 (242)
T ss_pred             CCCCCCCCCCCCCCCCCCCCCCCCCCCCCcccccCccceeceecCCCCeeccCCCeEEEEceEEEEEeecCCCCceeeeE
Confidence            0000        000000         00000            0000000000011111111111110       0011


Q ss_pred             echHHHH-------------HHHHHHHhccceEEehheEEEEECCccCCCCCceEEEEEEecCCCccccccccceEEEEe
Q Q_5943908_1      45 TGQHKMM-------------LLLSSALRIGSVHMDVSRILVKWCPYITPNMNTTIGITIKNNHHDDMSNINDMSTYISVK  111 (192)
Q Consensus        45 l~str~~-------------L~l~sll~~~s~Ei~IkkI~i~WCP~VEP~~~ssI~I~v~Y~~~~~~~~~~~D~tvv~~~  111 (192)
                      .+.-..+             -+|..+. ..=-...+.++.+.|-|.+--.....|.+-+.|+..+..-. + -..+....
T Consensus        81 ~i~P~~~~~~~~~~~f~~~~p~L~~iA-~~y~kyr~~~l~~~y~p~~~Ttt~G~v~m~~~~D~~d~~p~-~-~~~~~~~~  157 (242)
T 4V99_Jj          81 PIIPRLSVPAGDKPIYSGSAPHLRTIG-SAFAIHRWRALSFEWIPSCPTTTPGNLVLRFYPNYSTETPK-T-LTDLMDSE  157 (242)
T ss_pred             eeccccCCCCCCCCccccCCchHHHHH-HheEEEEEeEEEEEEeeCCCCCCCeEEEEEEecCCCCCCCC-C-HHHHhcCC
Confidence            1111111             2222222 22234555666666666666555556666555554443110 0 01122222


Q ss_pred             eeec----ccceeE--EE--eCCeeE-e----------ecCCeEEEceEEEEecCCeeccCCCcceeeEEEEEEEeeeec
Q Q_5943908_1     112 GKMS----EALQIT--WH--PASTLV-Y----------KKGMSCIFPWVVDVDTGSTEQESGSPALGEIKIWCYFKMQYH  172 (192)
Q Consensus       112 G~iS----E~L~Vv--i~--Pt~~li-~----------~~~~a~~lPWsV~vetd~~~~~e~~~~lG~lkiWC~~~~~~~  172 (192)
                      |..+    +...+.  +.  ++.+.+ +          ..+..+.-|=.+-+-+++.   ..+..+|.  |||.+++...
T Consensus       158 ~~~~~~vw~~~~~~~~l~~~~~~~~~~~~~~~~~~~~~~~d~~~~~~G~~~v~~~g~---~~~~~~G~--l~v~Y~V~l~  232 (242)
T 4V99_Jj         158 SLVLVPSLSGKTYRPKIETRGNPPELRNIDATAFSALSDEDKGDYSVGRLVVGSSKQ---AVVIQLGL--LRMRYSAEMR  232 (242)
T ss_pred             CceeecCCCCcccccccCCCCCCCeeeecCcccccCCCcccccceeeEEEEEEEcCC---CCcceeEE--EEEEEEEEEE
Confidence            2211    111110  00  000111 0          0111222222222222222   12223333  3555555554


Q ss_pred             CCCCcccccc
Q Q_5943908_1     173 KPSTRHIARA  182 (192)
Q Consensus       173 ~~~~k~~s~~  182 (192)
                      .+..-.-...
T Consensus       233 ~P~~~~~~~~  242 (242)
T 4V99_Jj         233 GATSISGVSA  242 (242)
T ss_pred             cceecCCCCC
Confidence            4443333322


No 12
>6MRL_B p41; Plant virus, Tombusvirus, CLSV, VIRUS; 3.2A {Cucumber leaf spot virus}
Probab=22.47  E-value=1.2e+02  Score=18.46  Aligned_cols=183  Identities=9%  Similarity=0.057  Sum_probs=89.7  Template_Neff=6.600

Q ss_pred             CCc----cccc--------ccC-------------CC-----CCCCCcCccee-EEEEEEcccCCcceEeeH--------
Q Q_5943908_1       1 MEG----LSSK--------AQT-------------MG-----REDDNRSSKMK-VFHSELVYGDNHNISIKK--------   41 (192)
Q Consensus         1 ~~~----~~~~--------~~~-------------~~-----~~~~~k~sK~~-ay~~~~i~g~~~n~~I~~--------   41 (192)
                      +.+    +..+        .++             .|     .....+| +|. .-.++.|++.++--.|..        
T Consensus        37 ~~~~~~~~~~~~~~~~~~~~~~~~~~~~~~~~~v~APvA~~~~vr~~~P-~~~~~~~~~~I~~rE~i~~V~~~s~~~~~~  115 (386)
T 6MRL_B           37 VDALGNVVSRATGRKKKSKGKEVQNQIVGGIGAIAAPVSITKRVRGMRP-SFRQTKGKVHIVHRELVTSVINLVGNFRVN  115 (386)
T ss_dssp             --------------------------------------------------------CBCCCEECCEEEEEECCSSCCEET
T ss_pred             hHhHHHHHHHHhhhccccCCCcccccccCCCcceecceeeeeeecCCCC-EEEecCCeEEEEceEEEEEEEeccCceecC
Confidence            000    0000        000             00     0001111 111 011122222222222222        


Q ss_pred             -------hheechH---HHHHHHHHHHhccceEEehheEEEEECCccCCCCCceEEEEEEecCCCcccc--ccccceEEE
Q Q_5943908_1      42 -------ADLTGQH---KMMLLLSSALRIGSVHMDVSRILVKWCPYITPNMNTTIGITIKNNHHDDMSN--INDMSTYIS  109 (192)
Q Consensus        42 -------~~fl~st---r~~L~l~sll~~~s~Ei~IkkI~i~WCP~VEP~~~ssI~I~v~Y~~~~~~~~--~~~D~tvv~  109 (192)
                             ..|.+.-   ..+-||..+. ..=-..++.++.+.|-|.+--.....|.+-..||-.+..-.  .+--..--+
T Consensus       116 ~g~~~~~~~~~inP~~~~tfpwLs~iA-~~y~kYr~~~l~~~yvp~~~TTt~G~V~ma~d~D~~D~~p~s~~~l~~~~~s  194 (386)
T 6MRL_B          116 NNVSAQIGQFRINPSNSSLFTWLPTIA-SNFDSYRFTSIRFVYVPLCATTETGRVSLFWDKDSQDPLPVDRAALSSYGHS  194 (386)
T ss_dssp             TBSSCCSSSCEECTTCTTTCSSTHHHH-TTCSEECCSCEEEEEEECSCTTCCCCEEEEEESSTTSCCCCSTTGGGSCSSE
T ss_pred             CCcceeeEEEEeCCCChhhCchHHHHH-hhceEEEEcEEEEEEEeCCCCCCCceEEEEEecCCCCCCCCCHHHHHccCCc
Confidence                   1122221   1234677666 45568899999999999999999999999999988764221  111235567


Q ss_pred             EeeeecccceeEEEeCCeeEeec-----CCeEEEceEEEEecCCeeccCCCcceeeEEEEEEEeeeecCCCCcccccccc
Q Q_5943908_1     110 VKGKMSEALQITWHPASTLVYKK-----GMSCIFPWVVDVDTGSTEQESGSPALGEIKIWCYFKMQYHKPSTRHIARAEI  184 (192)
Q Consensus       110 ~~G~iSE~L~Vvi~Pt~~li~~~-----~~a~~lPWsV~vetd~~~~~e~~~~lG~lkiWC~~~~~~~~~~~k~~s~~y~  184 (192)
                      +.+.+.+...+.|-.....-|-.     |.++.=|=.+.+-+++..   ....+|+  +|+.+++....+......-++-
T Consensus       195 ~s~~~w~~~~l~Ip~d~~~ry~~~~~~~D~kl~d~G~~~iat~g~~---~~~~~G~--L~v~Y~VeL~~P~~~~~~~~~~  269 (386)
T 6MRL_B          195 NEGPPWAETTLNVPTDGKQRFVTDSNTTDRKLVDLGQFAFATYAGG---SNNQIGD--IYVEYGVEFSEAQPAGGLTQYI  269 (386)
T ss_dssp             EEECTTSCEEEEECCCSCCEECCCSSCSSCSSSCSEEEEEEECCCS---CCSEEEE--EEEEEECCCBSCCCCCCCCCCE
T ss_pred             EEeCCccceEEEcCCCCcceeEeCCCCCCccceecEEEEEEEEeCC---CCCeeEE--EEEEEEEEEEeceeCCcchheE
Confidence            78888888887775443333332     223333455666666542   2456666  5888888777776555444411


Q ss_pred             CCeeeecC
Q Q_5943908_1     185 APSIEWGN  192 (192)
Q Consensus       185 ~P~i~WsN  192 (192)
                        +...+.
T Consensus       270 --~~~~~~  275 (386)
T 6MRL_B          270 --TKSVGA  275 (386)
T ss_dssp             --EEESSS
T ss_pred             --EEeecC
Confidence              112221


No 13
>2TBV_B TOMATO BUSHY STUNT VIRUS; VIRUS, Icosahedral virus; 2.9A {Tomato bushy stunt virus} SCOP: b.121.4.7
Probab=20.10  E-value=1.4e+02  Score=18.11  Aligned_cols=183  Identities=9%  Similarity=0.068  Sum_probs=82.8  Template_Neff=6.500

Q ss_pred             CCc---ccccccC-------------------------CC-----CCCCCcCccee--EEEEEEcc-cCCcceEeeH---
Q Q_5943908_1       1 MEG---LSSKAQT-------------------------MG-----REDDNRSSKMK--VFHSELVY-GDNHNISIKK---   41 (192)
Q Consensus         1 ~~~---~~~~~~~-------------------------~~-----~~~~~k~sK~~--ay~~~~i~-g~~~n~~I~~---   41 (192)
                      ++|   +..|..+                         .|     .-...+| +|.  .-..+.|+ +.++--.|..   
T Consensus        42 ~~~~~~~~~~~~~~~~~~~~~~~~~~~~~~~~~~~~v~APvA~~~~vr~~~P-~~~~~~~~~v~I~~~rE~i~~V~~s~~  120 (387)
T 2TBV_B           42 LQSAVGLGKKALNKVRNRRKQGNQQIITHVGGVGGSIMAPVAVSRQLVGSKP-KFTGRTSGGVTVTSHREYLTQVNNSSG  120 (387)
T ss_dssp             -------------------------------------------------------------CCCEECCEEEEEEEECCSS
T ss_pred             HhcchhHHHHHHHHHhhccccCCcceeeecCCCCceeecceeeeeeecCCCC-eEeecCCCeEEEecCeEEEEEeeeCCC
Confidence            111   0000000                         00     0000011 111  00112222 2222111211   


Q ss_pred             ---------hheechHH---HHHHHHHHHhccceEEehheEEEEECCccCCCCCceEEEEEEecCCCcccc--ccccceE
Q Q_5943908_1      42 ---------ADLTGQHK---MMLLLSSALRIGSVHMDVSRILVKWCPYITPNMNTTIGITIKNNHHDDMSN--INDMSTY  107 (192)
Q Consensus        42 ---------~~fl~str---~~L~l~sll~~~s~Ei~IkkI~i~WCP~VEP~~~ssI~I~v~Y~~~~~~~~--~~~D~tv  107 (192)
                               ..|.+.-.   .+-||..+. ..=-+.++.++.+.|-|.+--.....|.+-+.||-.+..-.  .+-...-
T Consensus       121 ~~~~~g~~~~~~~inP~~~~tfpwLs~iA-~~yekYr~~~l~~~y~p~~stTt~G~V~ma~d~Ds~D~~p~s~~~l~~~~  199 (387)
T 2TBV_B          121 FVVNGGIVGNSLQLNPSNGTLFSWLPALA-SNFDQYSFNSVVLDYVPLCGTTEVGRVALYFDKDSQDPEPADRVELANFG  199 (387)
T ss_dssp             EEEGGGCCSSTTCCSSCCTTSCSSGGGTT-TSBSEEEEEEEEEEEEESSCTTCCCCEEEECCSCTTSCCCCSHHHHTTCT
T ss_pred             ceecCceeeeEEEeCCCCcccCchHHHHH-HheeEEEEcEEEEEEEcCCCCCCCceEEEEEeCCCCCCCCCCHHHHHccC
Confidence                     12222211   234666666 45568999999999999999999999999998887663211  1111344


Q ss_pred             EEEeeeecccceeEEEeCCeeEeecCC-----eEEEceEEEEecCCeeccCCCcceeeEEEEEEEeeeecCCCCcccccc
Q Q_5943908_1     108 ISVKGKMSEALQITWHPASTLVYKKGM-----SCIFPWVVDVDTGSTEQESGSPALGEIKIWCYFKMQYHKPSTRHIARA  182 (192)
Q Consensus       108 v~~~G~iSE~L~Vvi~Pt~~li~~~~~-----a~~lPWsV~vetd~~~~~e~~~~lG~lkiWC~~~~~~~~~~~k~~s~~  182 (192)
                      -.+.+.+++.+.+.|.......|-.+.     ++.=|=.+.+-+++.   .....+|+|  |+.+++....+..-....+
T Consensus       200 ~~~s~~~w~~~~l~I~~d~~~~y~~~~~~~D~kl~d~G~f~iat~g~---~~~~~~GeL--~v~Y~Vel~~P~~~~~~~~  274 (387)
T 2TBV_B          200 VLKETAPWAEAMLRIPTDKVKRYCNDSATVDQKLIDLGQLGIATYGG---AGADAVGEL--FLARSVTLYFPQPTNTLLS  274 (387)
T ss_dssp             TCEEECSSSCEEEECCCCCCCEECSSSCCSCTTTTCCCCCEEEEESC---SSSCEEEEE--EEEEEEEECSBCCCCCCSE
T ss_pred             CcEEeCCccCEEEEccCCCcceeeeCCCCccccccccEEEEEEEecC---CCCCceEEE--EEEEEEEEEccccCccccc
Confidence            567888888888777554433333321     111122222333332   124567775  7777777766654433222


Q ss_pred             ccCCeeeecC
Q Q_5943908_1     183 EIAPSIEWGN  192 (192)
Q Consensus       183 y~~P~i~WsN  192 (192)
                      ...  +..++
T Consensus       275 ~~~--~~~~~  282 (387)
T 2TBV_B          275 SKR--LDLTG  282 (387)
T ss_dssp             EBC--BCSSS
T ss_pred             cee--eecCc
Confidence            222  11111
