# Supplementary material for: Emergence of two distinct spatial folds in a pair of plant virus proteins encoded by nested genes
Source: J Biol Chem. 2024 Mar 24;300(5):107218. doi: 10.1016/j.jbc.2024.107218 (PMC11044054; doi:10.1016/j.jbc.2024.107218)
Supplement: Supporting File S2 [file mmc9.rtf]

Pelargonium necrotic spot virus QWC36212.1
Artichoke mottled crinkle virus NP 039811.1
Carnation Italian ringspot virus ACX53273.1
Carnation Italian ringspot virus ACX53283.1
Carnation Italian ringspot virus ALJ30186.1
Carnation Italian ringspot virus NP 612583.1
Carnation Italian ringspot virus BCH68494.1
Cucumber Bulgarian latent virus NP 835256.1
Cucumber necrosis virus NP 040956.1
Cymbidium ringspot virus NP 613263.1
Cymbidium ringspot virus QVX32683.1
Eggplant mottled crinkle virus YP 008999614.1
Gentian virus A BBD13997.1
Grapevine Algerian latent virus AHZ12759.1
Grapevine Algerian latent virus QYA72593.1
Grapevine Algerian latent virus YP 002308432.1
Havel River virus YP 009507821.1
Lisianthus necrosis virus AAY98783.1
Moroccan pepper virus AFM91107.1
Moroccan pepper virus BAN92403.1
Moroccan pepper virus YP 009037609.1
Neckar River virus UBZ25482.1
Pelargonium leaf curl virus YP 009259670.1
Pelargonium necrotic spot virus NP 945117.1
Sitke waterborne virus BCH36665.1
Sitke waterborne virus BCH36670.1
Tomato bushy stunt virus AAT67234.1
Tomato bushy stunt virus CAB56482.1
Tomato bushy stunt virus NP 062900.1
Tomato bushy stunt virus QOP59260.1
Tomato bushy stunt virus QVX32678.1
Tomato bushy stunt virus QYA72475.1
Tombusviridae sp. QXV86641.1
